# WhatsApp-Based Intervention for Diabetes Prevention and Care in Argentina: Implementation and Process Evaluation

**DOI:** 10.2196/81098

**Published:** 2025-12-01

**Authors:** Analia Nejamis, Laura Gutierrez, María Victoria López, Alvaro Daniel Ciganda Cha, Omayra Mendoza Quispe, Vilma Irazola, Andrea Beratarrechea

**Affiliations:** 1 Department of Research in Chronic Diseases Institute for Clinical Effectiveness and Health Policy (IECS) Buenos Aires Argentina; 2 Institute for Clinical Effectiveness and Health Policy (IECS) Buenos Aires Argentina; 3 Faculty of Medicine National University of San Marcos Lima Peru; 4 Instituto de Medicina Traslacional e Ingeniería Biomédica (CONICET) – Instituto Universitario Hospital Italiano (IUHI) – Hospital Italiano de Buenos Aires (HIBA) Buenos Aires, null Argentina

**Keywords:** diabetes, prediabetes, mHealth, WhatsApp, text-message intervention, process evaluation, Latin America, primary care, implementation science, mobile phone

## Abstract

**Background:**

In Argentina, diabetes is a growing public health concern, with a prevalence of 14% in 2024 and projections reaching 15.4% by 2050. In this context, a Diabetes Prevention and Care Program was implemented in low-income areas across 3 provinces. A key component of the program was a WhatsApp (WhatsApp LLC)-based intervention aimed at promoting self-care, encouraging healthy behaviors, and supporting follow-up among people with diabetes, those at risk, and pregnant women.

**Objective:**

This study aimed to describe the implementation and process evaluation of a WhatsApp-based intervention within Argentina’s public health system, using the Carroll Implementation Fidelity Framework, focusing on challenges encountered, implementation strategies used, and lessons learned across the 3 target populations.

**Methods:**

The intervention was implemented in 40 primary care centers. The population included adults residing in the catchment areas of the selected primary care centers. Participants included adults with type 2 diabetes, people at moderate or high risk based on the Finnish Diabetes Risk Score, and pregnant women. A set of 192 educational and reminder messages was developed and validated through expert input and community feedback. Messages were tailored to each target population and delivered through WhatsApp via Twilio (Twilio Inc) Business API (application programming interface). We assessed implementation fidelity focusing on adherence to the intervention, participant responsiveness, quality of delivery, and contextual barriers.

**Results:**

A total of 11,029 participants were enrolled in this study, of whom 9983 (90.5%) had a valid mobile phone number registered in the system. Among these, 32.8% (3276/9983) had a diagnosis of type 2 diabetes, 53.3% (5320/9983) were identified as being at moderate or high risk based on the Finnish Diabetes Risk Score questionnaire, and 13.9% (1387/9983) were pregnant women. Overall, 67.3% (n=5749) opted in to receive messages, with the highest acceptance among those with diabetes (n=2169, 74.3%) and the lowest among at-risk people (n=2935, 62.1%). Message adherence was high: 88.7% (n=5004) of participants received at least the minimum number of educational messages expected, and the mean proportion of messages read per participant was 82.2% (SD 29.8). The dropout rate was low (6.1%) but higher among pregnant participants (14.6%). Message delivery issues mostly included problems with WhatsApp on the mobile phones of participants. Technical challenges, including server overload, were addressed during implementation.

**Conclusions:**

The WhatsApp-based intervention was feasible and well-received in public primary care settings in Argentina, particularly among people with diabetes. The experience illustrates how a WhatsApp-based intervention can be leveraged to strengthen service delivery in low-resource contexts, while also highlighting the need for further work on integration with electronic health records, tailoring of content to population needs, and strategies to enhance digital inclusion for underserved populations.

## Introduction

### Background

Diabetes mellitus remains one of the leading causes of mortality worldwide and represents a significant public health challenge. In 2024, an estimated 589 million adults (11.1% of the global population aged 20-79 years) were living with diabetes, and this figure is projected to rise to 853 million (13%) by 2050 [1]. Notably, around 95% of this increase is expected to occur in low- and middle-income countries [1]. The global burden is primarily driven by type 2 diabetes (T2D), which accounts for more than 90% of diagnosed cases and is associated with adverse cardiovascular, metabolic, maternal, and neonatal outcomes [1].

In Argentina, the prevalence of diabetes mellitus increased from 5.4% in 2021 to 14% in 2024, with estimates suggesting it may reach 15.4% by 2050 [1]. Combined with high levels of obesity (25.3%) and physical inactivity (44.2%), this trend highlights the urgent need to enhance prevention, early detection, and chronic disease management across the national health system [1,2]. The prevalence of gestational diabetes is estimated at 9.8% [3], further underscoring the national public health concern.

Considering that over 40% of the Argentine population relies exclusively on the public health care system [4], several initiatives have sought to strengthen T2D and gestational diabetes management at the primary care level. Strategies such as health care team training, patient education, scheduled follow-ups, and reminder phone calls have shown improvements in metabolic control and service use, particularly when both health care providers and patients were involved [5,6]. Other multicomponent programs have combined team training with educational outreach visits and digital health strategies, improving access to laboratory tests and ophthalmologic care, blood pressure control, and follow-up visits at 12 months [7]. Despite these efforts, sustaining effective strategies over time remains challenging, especially in resource-constrained primary care settings [8-10].

Mobile phones are now widely used to deliver health information remotely, often through text messaging [11]. This modality provides people with accessible health information and practical guidance to support daily self-management of their health. Given its relatively low cost, potential scalability, and the fact that text messaging does not require advanced technological skills, mobile communication offers a promising way for public health systems seeking to improve care for people living with chronic diseases [12]. These digital tools provide practical and cost-effective alternatives to conventional health promotion strategies, and studies evaluating these interventions generally report high feasibility and acceptability [13].

Argentina has high mobile phone and internet connectivity: 92% of the population uses a mobile phone and 86% uses the internet (Q4 2021), placing the country among the most digitally connected in Latin America [14,15]. WhatsApp is also widely used; 86% of internet users report weekly use in 2021 [16]. This high level of digital connectivity stands in contrast to Argentina’s persistent social inequalities, where nearly 40% of the population currently lives below the poverty line [17]. As a result, mobile technology is deeply integrated into daily life, particularly through WhatsApp, the most widely used and culturally accepted messaging platform in the country, offering a feasible channel for delivering health interventions.

### WhatsApp-Based Intervention as Part of a Diabetes Prevention and Care Program

Between 2021 and 2024, a Diabetes Prevention and Care Program was implemented within Argentina’s public health system, targeting low-income areas of 3 locations in Argentina: Salta, San Juan, and Tandil (Province of Buenos Aires). The program included training for health care teams, as well as screening and care strategies for 3 target populations: people with diabetes, those at high risk of developing the disease, and pregnant women.

A key component of the program was a WhatsApp-based intervention that delivered educational messages aimed at enhancing knowledge, promoting healthy lifestyle changes, supporting medication adherence, and functioning as motivational prompts to enhance self-management behaviors [18].

Using WhatsApp via business APIs (application programming interfaces) allows us to pair a familiar channel with delivery and read status indicators that enable auditable reach, and to implement an explicit, channel-specific opt-in to document and reconfirm consent. This supports a minimally intrusive, scalable approach to public health messaging within the public primary-care system. This study aimed to describe the implementation and process evaluation of this WhatsApp-based intervention within Argentina’s public health system, using the Carroll Implementation Fidelity Framework [19], focusing on the challenges encountered, strategies used, and lessons learned across 3 target populations.

## Methods

### Setting and Population

This study was conducted between 2021 and 2024 in 3 urban settings of Argentina: Salta (capital city of Salta Province, northwest region), San Juan and Gran San Juan (covering 4 of the 5 health zones of San Juan Province, Cuyo region), and Tandil (a midsized city in Buenos Aires Province).

A total of 40 primary care centers (PCCs) were included. Each PCC is responsible for a geographically defined catchment area, composed mostly of low-income neighborhoods where residents rely primarily on public health services. Eligible PCCs had to be located in socioeconomically disadvantaged areas, report at least 800 monthly outpatient visits, use community health workers (CHWs), and provide free medications through the national and provincial essential medicines supply program. PCCs were selected in coordination with local health authorities to ensure comparability with other centers in each city.

The population included adults residing in the catchment areas of the selected PCCs. Eligibility criteria included adults (aged 18 years or older) with T2D, people at moderate to high risk of developing T2D, and pregnant women. Participants were recruited through two complementary approaches: (1) opportunistic recruitment at PCCs, when individuals attended for routine visits or other health reasons; and (2) community-based recruitment, conducted by trained health workers through door-to-door household visits within the PCC catchment areas and community outreach activities (eg, neighborhood health fairs and community centers). CHWs used standardized screening forms, and received prior training on recruitment protocols, and field activities were supervised by PCC coordinators to ensure consistency across sites.

### Data Collection

Trained health care workers administered the validated Finnish Diabetes Risk Score (FINDRISC) questionnaire which was used to screen for people at moderate or high risk of developing T2D [20]. Data were collected using an electronic form developed in REDCap (Research Electronic Data Capture; Vanderbilt University) [21,22]. CHWs and nurses, previously trained to use the system, gathered sociodemographic and clinical information, including age, gender, cardiovascular risk factors, diabetes history, lifestyle behaviors (eg, fruit and vegetable intake and physical activity), and current treatments. Anthropometric measurements, such as height, weight, and abdominal circumference, were also collected to calculate diabetes risk using the FINDRISC tool integrated into the system.

Participants with moderate or high risk of T2D, or with diabetes, or pregnant, were enrolled in this study.

### Messaging System

#### Content Generation and Validation

A total of 192 tailored text messages were developed and validated: 40 for pregnant women, 87 for people with T2D, and 65 for those with moderate or high risk of developing T2D. Messages were categorized into key thematic categories, including general information, lifestyle modification, medication adherence, diabetes care, foot care, insulin use, hypoglycemia prevention, and follow-up reminders.

Content related to behavior change was developed based on principles of the Transtheoretical Model [23], which proposes 5 stages: precontemplation, contemplation, preparation, action, and maintenance. These were grouped into 3 stages, and messages were designed to align with the likely needs of each stage (eg, raising awareness in earlier stages, reinforcing motivation and skills in intermediate stages, and supporting long-term maintenance). A prior validation was conducted and included formative research through cognitive interviews with community members, expert review by local specialists and communicators, and pilot testing using a 7-item questionnaire to evaluate understanding, appeal, and suggestions for improvement. Messages related to diabetes care were adapted from a previous study of our team in Argentina and have shown good acceptability in people with diabetes living in low-resource settings [24-26]. Messages for pregnant women were developed with input from experts and local health authorities to ensure appropriateness and relevance.

A full list of validated messages is available in Multimedia Appendix 1.

#### Implementation Process

**Figure 1:**
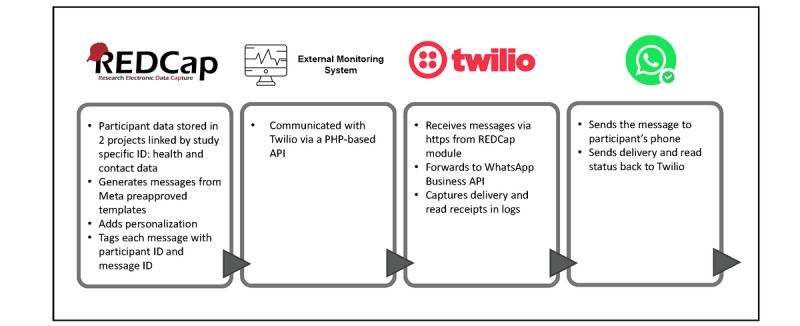
Data flow diagram of the WhatsApp-based messaging system. API: application programming interface; REDCap: Research Electronic Data Capture.

The messaging system followed an automated and organized sequence of steps for collecting participant data, creating messages, and delivering them. As shown in Figure 1, the process started with registering participant information in the REDCap data management platform. Once the forms were completed and all required fields validated, messages were generated using predefined algorithms implemented as an external module in a separate REDCap project. These algorithms considered the specific population subgroup to which the participant belonged, such as people with T2D (with or without insulin dependence), people with a moderate or high risk of developing T2D, or pregnant women classified by gestational week.

An ad hoc monitoring system operated as an intermediary between REDCap and the messaging system (Twilio). It performed automated daily queries of the REDCap database at scheduled intervals to identify pending messages for delivery. When applicable, these messages were transmitted to Twilio, which routed them to WhatsApp for final delivery to participants. Each step in the process was recorded systematically, including the delivery status of each WhatsApp message. Specific reports were generated for monitoring throughout this study to ensure the intervention was operating as intended.

#### Delivery

Once the REDCap forms were completed and validated, the system automatically created the messages and initiated their delivery. The first message served as an opt-in request, asking participants to confirm their consent to receive messages as part of this study. Only participants who agreed to this initial message were included in the text messaging system. After confirmation, each participant received a welcome message, followed by educational, tailored content based on their population group. Pregnant women received messages according to their gestational week, continuing through the expected duration of their pregnancy. All messages were personalized using the participant’s first name. Additionally, regional customization was incorporated into the welcome messages, including province-specific references to health authorities, such as the names of the local diabetes programs or ministries of health.

### System Requirements

The following components were required to operate the messaging system: (1) data repository: REDCap was used to store participant information, message templates, and the algorithms for message delivery; (2) Twilio and WhatsApp integration: a verified Meta Business account was required to configure and authorize the WhatsApp Business API via Twilio; (3) cell phone lines: dedicated local numbers were acquired through Twilio to enable automated message delivery via WhatsApp; (4) preapproved message templates: WhatsApp mandated that all message templates be reviewed and approved before deployment; and (5) API-based communication: the control system communicated with Twilio via a PHP-based API, while Twilio interacted with the control system via webhooks.

### Data Management and Security

As illustrated in Figure 1, each message was generated in REDCap through an external module that applied Meta-approved templates. Templates included a common header, a personalized greeting with the participant’s first name, and the educational content scheduled for that week. Each message was tagged with both a participant ID and a message ID.

Delivery and read receipts were retrieved from Twilio logs and matched to participants using phone number and message text. These logs were consolidated weekly through R (R Project for Statistical Computing; R Foundation) scripts to cover the full study period.

Contact details were stored in a separate REDCap project from health data, and linkage was made only via a study-specific participant ID. Phone numbers were retained solely to enable message delivery.

Access to Twilio logs was restricted to account administrators, who downloaded them to a secure shared folder for statistical analysis. Twilio retained logs of up to 10,000 lines (≈3-6 months of activity), which were then consolidated. Data transfers were encrypted in transit (https). Data were stored on cloud servers without encryption at rest. No additional procedures were implemented to minimize exposure of phone numbers, which were necessary for message delivery.

Participants retained the right to withdraw at any time, either by replying with an opt-out request or by directly contacting this study’s team using the information provided in the consent form.

### Evaluation Framework

The evaluation was based on Carroll Implementation Fidelity Framework [19], which assesses both the implementation process and the extent to which participants received the intervention as intended (Table 1).

This framework has been widely applied in previous studies [27-29] to evaluate the consistency and quality of intervention delivery. It focuses on key domains such as adherence, quality of delivery, participant responsiveness, and contextual factors.

In our process evaluation, using data collected at various stages of the intervention, adherence was examined by determining whether the planned activities were carried out as intended. This included verifying that all key messages, including opt-in (ie, initial confirmation of consent to receive weekly messages), welcome, weekly, and final messages, were successfully generated and delivered to participants. Additionally, we analyzed the coverage of the intervention across the 3 participant subgroups: people with T2D, those at moderate or high-risk of developing diabetes, and pregnant participants.

Participant responsiveness was measured by calculating the opt-in acceptance rate. We also tracked the proportion of messages that were read by participants relative to those delivered. Participants’ withdrawal from the intervention was also documented.

Contextual factors that may have impacted implementation were also considered, including technical issues and challenges reported by participants.

**Table 1:** Fidelity dimensions and indicators.

Dimensions		Research questions	Indicators
Adherence	Was the WhatsApp-based intervention implementation as planned?		Opt-in messages generated: proportion of participants with an opt-in message generated/participants who had a valid cellphone number. Welcome message generated: proportion with a welcome message generated/participants who accepted the opt-in message. Final message generated: proportion with a final message generated/participants who completed the intervention.
Adherence	Did the intervention reach all target populations?		Proportion of participants with a valid cellphone number. Opt-in messages delivered: proportion receiving the opt-in message/participants with an opt-in message generated. Welcome messages delivered: proportion receiving the welcome message/participants with a welcome message generated. Educational messages delivered (minimum expected): proportion receiving at least the minimum number of educational messages expected/participants who accepted the opt-in message. Educational messages delivered (average): average number of educational messages received per participant. Final messages delivered: proportion receiving the final message/participants with a final message generated.
Participant responsiveness	What was the level of participant engagement?		Acceptance rate of opt-in messages: proportion who accepted the opt-in message/participants who received the opt-in message. Messages read^a^: proportion of delivered messages that were read/messages sent. Dropout rate: proportion of participants who withdrew from the intervention.
Quality of delivery	Were delivery errors minimized?		Proportion of message delivery failures.
Contextual factors	What barriers affected implementation?		Description of technical issues and participant-related challenges.

^a^Indicate message opening.

### Data Analysis

Descriptive statistics were used to summarize sociodemographic and clinical characteristics overall and by target group, and to describe implementation fidelity indicators. Group comparisons assessed differences in access (valid cellphone number) and acceptability (opt in). As a sensitivity analysis, multivariable logistic regression models including sex, age, educational attainment, and comorbidity category were fitted for each outcome; odds ratios with 95% CIs are reported in Table S1 in Multimedia Appendix 2 and Table S1 in Multimedia Appendix 3.

### Ethical Considerations

This study was reviewed and approved by 3 institutional ethics committees in Argentina: the Institutional Review Board for Research Studies (Consejo Institucional de Revisión de Estudios de Investigación) of the Hospital Privado de Comunidad in Mar del Plata, Province of Buenos Aires (approved date: July 7, 2022); the Research Ethics Committee of the Ministry of Public Health of San Juan (May 12, 2021); and the Provincial Commission for Biomedical Research of the Ministry of Public Health of Salta (March 8, 2021).

Participation in this study was voluntary, and written informed consent was obtained from all participants during the screening process. Consent covered both study data collection and the receipt of digital messages. Participants confirmed their enrollment by explicitly opting in to the messaging system. They retained the right to withdraw at any time, either by replying directly to the system or by contacting this study’s team using the information provided in the consent form. Participants did not receive financial or material compensation for their participation in this study, and no identifiable images of individual participants are included in this paper or its supplementary materials.

Confidentiality was strictly maintained, and all procedures complied with Argentina’s Personal Data Protection Law (Ley 25.326). The use of WhatsApp Business API required attention to privacy and data protection. Messages were sent using standardized templates and contained no personal health information. Participant data were stored in REDCap with password-protected access. While messages were transmitted over encrypted https connections, data were stored on secure cloud servers.

## Results

### Participant Characteristics

**Figure 2:**
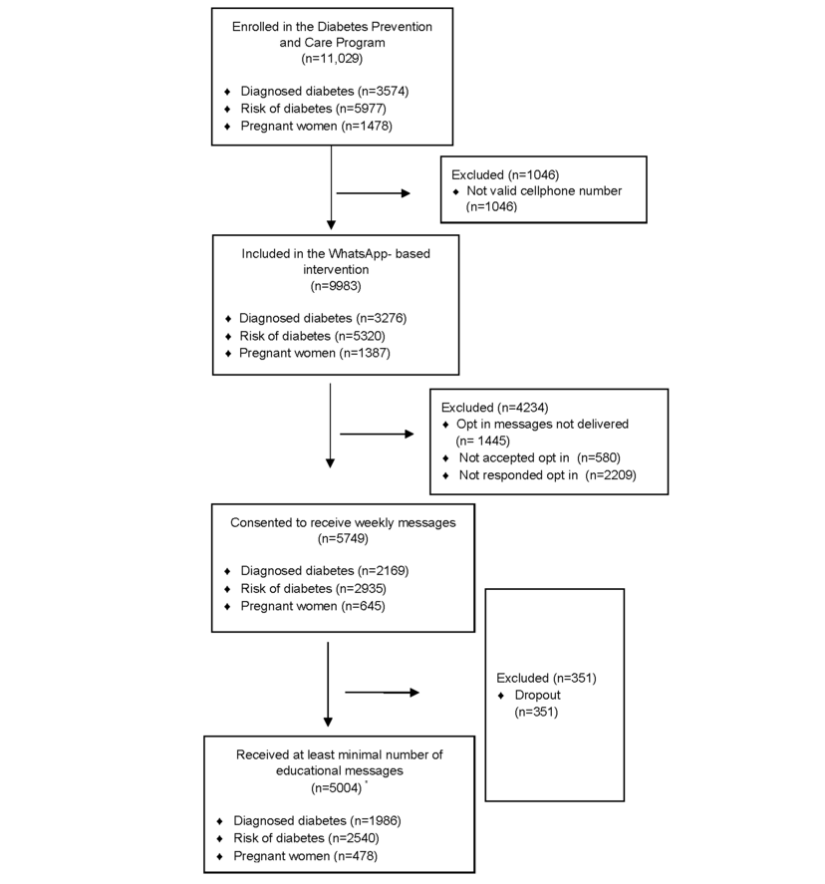
Participants flow diagram. *Among the 5749 participants who consented, 5004 reached the minimum message dose; 745 did not. Dropouts (n=351) occurred independently of reaching the minimum dose.

A total of 11,029 participants were enrolled in this study, of whom 9983 (90.5%) had a valid mobile phone number registered in the system. Among these, 32.8% (3276/9983) had a diagnosis of T2D, 53.3% (5320/9983) were identified as being at moderate or high risk based on the FINDRISC questionnaire, and 13.9% (1387/9983) were pregnant women (Figure 2).

Table 2 summarizes the main sociodemographic and clinical characteristics of participants enrolled in the Diabetes Prevention and Care Program.

Most participants were women (n=7725, 77.4%), with a mean age of 44.5 (SD 15.6) years. Participants with a valid cellphone number tended to be slightly younger compared to those who could not be reached. Age distribution also varied across target groups, from a mean of 51.9 (SD 13.6) years among people with diabetes to 26.4 (SD 5.9) years among pregnant participants.

More than half of all participants (n=5332, 53.4%) had completed or partially completed secondary education. Among those with a valid cellphone number, a higher proportion had completed secondary education, whereas among participants without a valid phone number, the majority had only completed primary education. In target groups, the proportion with secondary education was higher among the pregnant women group (1008/1387, 72.7%). In contrast, people with diabetes showed lower educational attainment, with 46.3% (1516/3276) having completed only primary education or less.

Comorbidities were common, affecting 76% (7584/9983) of participants overall. Those with a valid cellphone number more frequently reported two or more conditions.

**Table 2:** Sociodemographic and clinical characteristics of participants enrolled.

Characteristics		Total	Valid cellphone number	
			Yes	No
Participants, N		11,029	9983	1046
Female, n (%)		8476 (76.9)	7725 (77.4)	751 (71.8)
Age (years), mean (SD)		45.0 (15.7)	44.5 (15.6)	49.6(16.9)
**Educational level^a^, n (%)**				
	Primary school	4004 (36.3)	3554 (35.6)	450 (43)
	Secondary school	5811 (52.7)	5332 (53.4)	479 (45.8)
	University or higher	927 (8.4)	858 (8.6)	69 (6.6)
	Not available	287 (2.6)	239 (2.4)	48 (4.6)
**Comorbidities^b^, n (%)**				
	None	2644 (24)	2395 (24)	249 (23.8)
	One condition	3629 (32.9)	3252 (32.6)	377 (36)
	Two or more conditions	4743 (43)	4327 (43.3)	416 (39.8)

^a^Includes complete and incomplete levels.

^b^Includes obesity, hypertension, hypercholesterolemia, and cardiovascular disease.

### Implementation Evaluation

#### Adherence to Intervention and Responsiveness

Of the 11,029 enrolled participants, 9983 (90.5%) had a valid cell phone number registered, necessary for intervention delivery. This proportion was highest among pregnant women (n=1387, 93.8%) and lowest among people at risk of diabetes (n=5320, 89%; Table 3).

Opt-in messages were successfully generated for 95.6% (n=9546) of participants with a valid cellphone number, with nearly complete coverage in the diabetes group (n=3253, 99.3%) and the at-risk group (5269/5320, 99%), but lower in the pregnant group (n=1024, 73.8%). Of the generated opt-in messages, 89.4% (n=8538) were successfully delivered, and 91.1% (n=7778) of those delivered were read. Reading rates were slightly higher in the diabetes group (2699/2918, 92.5%) and showed similar levels across groups.

A total of 67.3% (n=5749) of participants accepted the opt-in message, while 25.9% (2209/8538) did not respond, and 6.8% (580/8538) declined to participate. Acceptance was highest among people with diabetes (n=2169, 74.3%), followed by the pregnant group (n=645, 72%) and those at risk of diabetes (n=2935, 62.1%).

Regarding weekly educational content messages, 88.7% (n=5004) of participants received at least the minimum number of messages expected, defined as 20 for people with diabetes and 12 for those at risk. Adherence was highest among the diabetes group (n=1986, 91.6%), followed by the at-risk group (n=2540, 86.5%), and lowest among pregnant participants (n=478, 74.1%), for whom the expected number of messages varied according to gestational week at the time of enrollment, and the mean proportion of messages read per participant was 82.2% (SD 29.8). Final messages were generated for 85.5% (n=1854) of participants in the diabetes group and 90.3% (n=2649) in the at-risk group and delivered in 93.2% (n=4199) of those cases.

The dropout rate among participants who had accepted the messaging intervention was 6.1%, with the lowest rate in the diabetes group (4.4%) and the highest among pregnant participants (14.6%).

**Table 3:** Indicators of adherence to intervention and responsiveness by target group^a^.

Indicators				Total		Diagnosed diabetes		Risk of diabetes		Pregnant women
Participants, n				11,029		3574		5977		1478
With a valid cellphone number, n (%)				9983 (90.5)		3276 (91.7)		5320 (89)		1387 (93.8)
Opt-in message generated, n (%)				9546 (95.6)		3253 (99.3)		5269 (99)		1024 (73.8)
Opt-in message delivered, n (%)				8538 (89.4)		2918 (89.7)		4724 (89.7)		896 (87.5)
Opt-in message read, n (%)				7778 (91.1)		2699 (92.5)		4270 (90.4)		809 (90.3)
Accepted opt-in message^b^, n (%)				5749 (67.3)		2169 (74.3)		2935 (62.1)		645 (72)
Welcome message generated, n (%)				5582 (97.1)		2077 (95.8)		2860 (97.4)		645 (100)
Welcome message delivered, n (%)				5318 (95.3)		1988 (95.7)		2724 (95.2)		606 (94)
Welcome message read, n (%)				4991 (93.9)		1887 (94.9)		2547 (93.5)		557 (91.9)
**Educational messages**										
	Received ≥ minimum expected messages^c^, n (%)		5004 (88.7)		1986 (91.6)		2540 (86.5)		478 (74.1)	
	**Number of messages delivered per participant**									
		Mean (SD)	34.2 (20.9)		45 (23.4)		30 (16.5)		19 (9.5)	
		Median (IQR)	27 (22-44)		40 (27-60)		25 (22-35)		19 (12-27)	
	**Messages read per participant out of delivered (%)**									
		Mean (SD)	82.2 (29.8)		83.6 (28.9)		81.2 (30.5)		82.3 (29.6)	
		Median (IQR)	100 (77-100)		100 (82-100)		100 (73-100)		100 (75-100)	
Final message generated^d^, n (%)				4503 (78.3)		1854 (85.5)		2649 (90.3)		—^e^
Final message delivered, n (%)				4199 (93.2)		1744 (94.1)		2455 (92.7)		—
Final message read, n (%)				3392 (80.8)		1477 (84.7)		1915 (78)		—

^a^Percentages were calculated sequentially, using the frequency in the preceding row as the denominator, unless otherwise specified.

^b^Denominator: opt-in messages delivered.

^c^Participants with diabetes who received at least 20 messages, and those at risk of diabetes who received at least 12 messages. For pregnant women, the number of messages calculated was based on the gestational week at the time of enrollment in this study.

^d^Denominator: opt-in messages accepted.

^e^Not available.

#### Participant Acceptability

Participant characteristics according to opt-in message acceptance are detailed in Table 4. Additional disaggregated data by target group are provided in Table S1 in Multimedia Appendix 4.

Among the 8538 people who received the opt-in message, 67.3% (n=5749) accepted, while 32.6% (2789/8538) either declined or did not respond. As a complementary analysis to evaluate the impact of initial noncoverage, when considering the total enrolled population as the denominator, the overall acceptance rate was 52.1% (5749/11,029) instead of 67.3% (n=5749) among those with a valid phone number.

A higher proportion of women accepted the intervention (n=4515, 78.5%) compared to those who did not accept (n=2057, 73.8%), while men represented 21.5% (n=1234) of those who accepted and 26.2% (n=732) of those who did not. The mean age was slightly lower among those who agreed to receive the messages (44.0, SD 14.6 years) compared to those who did not (46.7, SD 16.0 years).

Among participants who accepted the intervention, a greater proportion had completed secondary school (n=3123, 54.3%) compared to primary school (n=1936, 33.7%). In the group that did not accept, the proportion with primary education (n=1105, 39.6%) was higher than among those who accepted. The prevalence of self-reported comorbidities was similar across groups, with 78.1% (4489/5749) among those who accepted and 77.8% (2170/2788) among those who did not.

In sensitivity analyses with adjusted models, older age was consistently associated with lower odds of both having a valid cellphone number and accepting opt-in, whereas multiple comorbidities were associated with higher odds. For the opt-in accepted outcome, male sex and lower educational attainment were also associated with reduced odds (Table S1 in Multimedia Appendix 2 and Table S1 in Multimedia Appendix 3).

**Table 4:** Participant characteristics by opt-in message acceptance.

Characteristics		Opt-in message accepted			
		Yes		No^a^	
Participants, N		5749		2789	
Female, n (%)		4515 (78.5)		2057 (73.8)	
Male, n (%)		1234 (21.5)		732 (26.2)	
Age (years), mean (SD)		44.0 (14.6)		46.7 (16.0)	
**Educational level^b^, n (%)**					
	Primary school		1936 (33.7)		1105 (39.6)
	Secondary school		3123 (54.3)		1399 (50.2)
	University or higher		571 (9.9)		202 (7.2)
	Not available		119 (2.1)		83 (3)
**Comorbidities^c^, n (%)**					
	None		1258 (21.9)		615 (22.1)
	One condition		1831 (31.8)		980 (35.1)
	Two or more conditions		2658 (46.2)		1190 (42.7)

^a^Includes those who declined or did not respond to the opt-in message.

^b^Includes complete and incomplete levels.

^c^Includes obesity, hypertension, hypercholesterolemia, and cardiovascular disease.

### Quality of Delivery

The most commonly reported delivery issues included phone numbers not enabled for WhatsApp, users who had not accepted WhatsApp’s terms of service, outdated or unsupported versions of the WhatsApp app, and improperly formatted phone numbers.

### Contextual Factors

Beyond individual delivery issues, the system faced broader contextual challenges. Some technical difficulties were encountered during the delivery of educational text messages via WhatsApp using the Twilio interface. At some point of this study, during periods of high system load, the server’s processing capacity was insufficient to manage the volume of data exchange until the server was upgraded. This occasionally resulted in delays in the registration of message delivery status, particularly when response signals were received before outgoing messages could be properly logged. Additionally, for pregnant women, some opt-in messages could not be generated due to missing or incorrectly coded gestational age data.

Additional limitations included suboptimal code performance and intermittent communication failures between the messaging control system and the REDCap platform. The underlying code was subsequently optimized to handle the increased message volume as more participants were enrolled.

To ensure accurate message tracking, Twilio logs were manually retrieved every week and matched with REDCap data using participant phone numbers.

## Discussion

### Principal Findings

This study examined the implementation of a WhatsApp-based intervention for people with T2D, moderate or high risk of developing this condition, and pregnant women, in the context of Argentina’s public health system, focusing on adherence, engagement, delivery quality, and contextual barriers. The intervention followed a structured delivery process, from opt-in messages to weekly health education content and final closing messages.

There is robust evidence that text messaging interventions can support chronic disease management, such as in T2D [30,31]. However, limited evidence exists on how these programs are implemented in routine primary care settings, particularly in low-resource environments [32]. Our findings contribute to addressing this gap by documenting the novel use of WhatsApp for digital health messaging in the real-world public health sector in Argentina, providing insights on feasibility, potential scalability, and engagement in resource-constrained settings.

While most messages were successfully delivered, a small proportion were affected by technical issues, such as invalid cellphone numbers (approximately 1046/11,029, 10%) or intermittent connectivity problems. Similar challenges have been reported in other mobile health interventions for chronic disease [27,33]. Steinman et al [33] reported that 40% of participants did not receive messages due to frequent changes in personal mobile numbers driven by cost-saving strategies, while Van Olmen et al [27] found that nearly one-third of project phones became unusable because of subscription cancellations, breakage, or loss, despite providing SIM cards and devices. Both studies also described infrastructural limitations, such as weak network coverage and recurrent software errors [33].

In comparison, the proportion of unreachable participants in our study was lower, suggesting that integrating WhatsApp within existing care structures, with validated contact information and proactive follow-up, may help mitigate some of these barriers. Nonetheless, the percentage of nonreached people highlights possible inequities: those who could not be reached tended to be older, more often male, and with lower educational levels compared to those successfully contacted. Although the differences were modest, these patterns reflect potential digital exclusion linked to socioeconomic and demographic disparities. These trends are consistent with prior evidence showing that older age is negatively associated with adoption of mobile health technologies, while higher educational attainment increases access and use [34]. These patterns could be related to the lower digital literacy, physical or cognitive limitations, and greater concerns about privacy for older adults, whereas people with lower education are more likely to experience restricted access to smartphones, unstable connectivity, or difficulties interpreting health messages. Taking into account the inequities in phone ownership, access, and use [35], future interventions should consider tailored recruitment strategies to ensure that no population is systematically excluded due to digital access limitations, such as providing guidance for phone access and registration, using complementary communication channels for those without WhatsApp, and improving the process of validating contact information.

Another critical component of implementation is participant engagement and acceptability. In this study, we assessed these aspects by examining the read rate of the initial opt-in message, the proportion of participants who agreed to receive weekly messages, the weekly message read rates during the intervention, and dropout rates over time. Engagement levels were high: approximately two-thirds of participants accepted the intervention, over 90% (7778/8538) read the opt-in message, weekly read rates averaged 82.2% (SD 29.8), and only 6.1% of participants dropped out. Our findings are consistent with previous research [24,31,36], showing that text messaging interventions are generally well-received by both participants and health care providers. The engagement observed in this study may be related to the intervention’s carefully designed structure, including its integration into routine primary care practice and the proactive efforts of health care teams to validate contact information.

These findings support the potential of WhatsApp as an effective platform to reach and engage broad populations and sustain participation, particularly when integrated into existing care structures. Compared to SMS, WhatsApp might offer advantages such as greater user familiarity, support for multimedia content, and faster message visibility, which could enhance engagement in low-resource settings.

Participant engagement levels differed across the 3 target groups. People with diabetes showed the highest acceptance and lowest dropout rates, likely reflecting a greater perceived need for ongoing support. Those at risk of diabetes had the lowest acceptance, while pregnant women showed higher acceptance of the messages than the at-risk group, but had a higher dropout rate. In this sense, although there is evidence that text messaging interventions can be effective during pregnancy, particularly for promoting healthy behaviors [37-40], the higher dropout rates observed in this group may reflect the competing demands pregnant women often face, such as medical appointments, childcare responsibilities and work, as well as potential preferences for more personalized messaging which may limit their ability to remain engaged with supplementary programs [41,42].

In our study, people identified as being at risk of developing diabetes were the least likely to accept the intervention. This group was, on average, younger than those with diabetes, which may have contributed to a lower perceived awareness, diminished need for support, and reduced motivation to engage with preventive interventions. These findings highlighted the importance of designing targeted strategies that enhance risk awareness and encourage participation in early-stage prevention efforts. Other studies have reported comparable trends, highlighting that engagement levels significantly impact long-term outcomes, particularly in prediabetes self-management programs [43,44].

Sociodemographic factors also influenced acceptability in our study, with men, older participants, and those with lower educational attainment consistently less likely to opt in across all target populations. These patterns underscore that even within targeted populations, digital interventions may reach participants unevenly, potentially reinforcing existing sociodemographic inequities. An analysis of barriers and facilitators of mobile health adoption among people with low socioeconomic position supports these trends [45], identifying multiple factors such as digital literacy, trust, and need for in-person support, that are critical for successful adoption and use. Similarly, a systematic review of mobile health use [34] found that women were more likely to adopt mobile health services than men, indicating that lower engagement among men may reflect differences in health-seeking behavior and health awareness. In light of these findings, tailored strategies that consider gender, age, and educational level, such as proactive outreach, simplified content, and personalized relevant messaging, may enhance relevance and comprehension and could help maximize engagement across all subgroups [35].

Another key aspect in our study was the resolution of technical barriers, particularly those related to the high volume of data processed. One of the main issues encountered was system overload due to the high rate of outgoing messages, which exceeded the initial server’s capacity. This led to delays in updating message delivery status, complicating the integration with the REDCap platform and obstructing real-time monitoring. To address these challenges, substantial improvements were implemented to both the server infrastructure and the system’s code. The server was upgraded with expanded memory and transitioned to a more robust disk configuration, increasing its capacity to handle large data volumes. In parallel, code optimizations, particularly in the communication protocols with REDCap, streamlined data processing and improved log matching. These enhancements strengthened the reliability of message delivery and tracking, reduced the need for manual interventions, and improved overall system efficiency.

Additionally, using WhatsApp via the Business API, a familiar and widely accessible platform in Argentina, minimized technical barriers. The API enabled automated logging of delivery and read statuses, and captured explicit, channel-specific opt-ins and opt-outs, providing auditable, high-fidelity process data. Together, these technical improvements contributed to the intervention’s feasibility.

### Limitations

This real-world implementation study demonstrated that WhatsApp-based text messaging interventions were successfully integrated into public health services; however, several limitations should be considered when interpreting these results.

First, while messages were tailored by participant subgroups (people with T2D, at-risk of diabetes, or pregnant participants), they were not customized at the individual level. This lack of individual tailoring may have limited the intervention’s acceptability and adherence, as more personalized content or follow-up could have increased relevance and engagement [11]. However, we implemented a local validation of the message’s content at the regional level and included data privacy warnings in some messages, thereby enhancing the contextual relevance and participant trust.

Second, the intervention included general reminder messages encouraging participants to attend medical appointments, but lacked an individualized reminder system. Evidence suggests that individualized reminders, with specific dates and times, can significantly improve attendance in primary care settings [31,46]. Future integration with electronic health records could enable automated, personalized notifications. Third, another limitation of this study is the potential for selection and participation bias. Participants without a valid cellphone number who could not be reached differed modestly in terms of age and educational level compared to those who could be reached. Participation in the intervention also depended on willingness to opt in, which may have led to the overrepresentation of individuals who are more digitally connected, engaged with the health system, or have higher health literacy. As a result, engagement estimates may not be fully generalizable. Future implementations should consider strategies to enhance digital inclusion and monitor differential participation from the outset. Additionally, randomized controlled trials could provide complementary high-quality evidence on intervention effectiveness.

Fourth, despite WhatsApp’s widespread use in Argentina, engagement with messages varied across participants. This suggests that high platform penetration alone does not guarantee acceptance or behavioral change. Future adaptations should consider factors such as message content, perceived relevance, and user trust to optimize engagement across different subgroups. Finally, the engagement metrics reported reflect process outcomes rather than behavioral or clinical impact. Changes in self-care practices or glycemic control are beyond the scope of this evaluation and will be analyzed separately alongside other components of the Diabetes Prevention and Care Program.

### Conclusions

This study evaluated the implementation of a WhatsApp-based text messaging intervention for diabetes prevention and care in 3 locations within Argentina’s public primary health care system. The intervention successfully reached key target groups (people with diabetes, individuals at risk, and pregnant women), demonstrating the feasibility of integrating digital messaging into routine care. However, engagement varied across participants, and several technical and contextual factors likely influenced message adherence and acceptance.

Integrating digital tools into primary care and adapting them to local contexts can strengthen service delivery, but future efforts should focus on improving interoperability with electronic health records, personalizing content by cohort and comorbidity profile, and ensuring equitable access for populations with limited digital connectivity.

## Data Availability

The datasets generated or analyzed during this study are available from the corresponding author on reasonable request.

## Appendix

Multimedia Appendix 1 List of validated text messages.

Multimedia Appendix 2 Sociodemographic characteristics associated with valid cellphone number in participants.

Multimedia Appendix 3 Sociodemographic characteristics associated with intervention acceptance (opt-in).

Multimedia Appendix 4 Participant characteristics by opt-in message acceptance and target group.

## Abbreviations

API: application programming interface
CHW: community health worker
FINDRISC: Finnish Diabetes Risk Score
PCC: primary care center
REDCap: Research Electronic Data Capture
T2D: type 2 diabetes
