# Supplementary material for: WhatsApp-Based Intervention for Diabetes Prevention and Care in Argentina: Implementation and Process Evaluation
Source: JMIR Form Res. 2025 Dec 1;9:e81098. doi: 10.2196/81098 (PMC12706442; doi:10.2196/81098)
Supplement: Multimedia Appendix 1 [file formative_v9i1e81098_app1.docx]

**For people with diabetes**

| **Number** | **Domains** | **Spanish version of the SMS** | **English translation** |
| --- | --- | --- | --- |
| 1 | General information | La DIABETES es una enfermedad que dura toda la vida (crónica). La falta de insulina o la dificultad para utilizarla hace que el azúcar de los alimentos aumente y se acumule en la sangre (azúcar alta en sangre). | DIABETES is a lifelong (chronic) disease. It happens when the body doesn’t make enough insulin or can’t use it properly, causing sugar from food to build up in the blood (high blood sugar). |
| 2 | General information | ¡La DIABETES es una enfermedad crónica, pero se puede controlar! Si estás controlado, vas a estar bien y llevar una vida normal. | DIABETES is a chronic disease, but it can be controlled! If it is controlled, you will be fine and lead a normal life. |
| 3 | General information | Las complicaciones que causa la DIABETES (como el infarto y la ceguera) pueden evitarse. ¿Cómo? Cuidándose. Chárlalo con tu médico. | The complications caused by DIABETES (such as heart attack and blindness) can be avoided. How? By taking care of yourself. Speak with your doctor. |
| 4 | General information | Si tenés DIABETES, es importante que tu presión arterial no sea mayor a "14/9". Así evitarás tener problemas en el corazón y en los riñones. | If you have DIABETES, it is important that your blood pressure is not higher than "14/9". This will help you avoid heart and kidney problems. |
| 5 | General information | La DIABETES a veces es silenciosa. Algunas personas no tienen síntomas y no se dan cuenta de que su azúcar está alta. ¿Te mediste el azúcar últimamente? | DIABETES is sometimes silent. Some people don't have symptoms and do not realize that their blood sugar is high. Have you tested your blood sugar recently? |
| 6 | General information | ¿Sabías que cuando el azúcar en la sangre está alto podés marearte, ver borroso, sentir cansancio y orinar más seguido? Si te pasa esto, consulta al médico. | Did you know that when your blood sugar is high, you can get dizzy, have blurry vision, feel tired, and urinate more often? If this happens to you, consult a doctor. |
| 7 | General information | Para que tu presión esté bien tenés que tener menos de 140/90 o 14/9. ¿Cuál es tu presión? | For your blood pressure to be at a healthy level, you must have less than 140/90 or 14/9. What is your blood pressure? |
| 8 | General information | Si tenés presión alta, anota tus valores de presión en un papel y llevalos a la consulta con el médico. Así puede saber cómo va el tratamiento. | If you have high blood pressure, write down your pressure readings on a piece of paper and bring them to your doctor’s appointment. This will help you and your doctor know how your treatment is going. |
| 9 | General information | Se puede llevar una vida normal teniendo DIABETES. Sólo es necesario comer sano, hacer ejercicios habitualmente y tomar los medicamentos que te da el médico. | You can live a normal life with DIABETES. You just need to eat healthy, exercise regularly, and take the medications your doctor gives you. |
| 10 | General information | ¿Sabías que los problemas que trae la DIABETES al corazón, los ojos y los riñones se pueden evitar? ¿Cómo? Manteniendo el azúcar en la sangre controlada. | Did you know that the problems that DIABETES brings to the heart, eyes, and kidneys can be avoided? How? By keeping blood sugar under control. |
| 11 | Diabetes care | Consulta con tu médico para chequear tus niveles de azúcar en sangre. ¡El diagnóstico se hace con un análisis muy simple! Aconsejale también a tus familiares que se midan el azucar. | Talk to your doctor about checking your blood sugar levels. The diagnosis is made with a very simple test! Advise your family members to test their blood sugar too. |
| 12 | Diabetes care | Para que tu DIABETES esté bien controlada tenés que tener un valor de hemoglobina glicosilada cercano a 7 en los analisis de sangre. ¿Cuál es el tuyo? | For your DIABETES to be controlled well, you must have a glycosylated hemoglobin value close to 7 in your blood tests. What is yours? |
| 13 | Diabetes care | Si tus valores de azúcar en sangre están altos, visita a tu médico. ¡Él ayudará a que bajen para que puedas estar bien! | If your blood sugar values are high, visit a doctor. They will help bring them down so you can be well! |
| 14 | Diabetes care | Si aprendes a cuidarte vas a estar mejor! Los talleres de DIABETES que se realizan en los centros de salud son de mucha ayuda. Acércate a tu centro de salud. | If you learn to take care of yourself, you will be better off! The DIABETES workshops held in health centers are very helpful. Come to your health center. |
| 15 | Diabetes care | ¿Sabías que los médicos de los centros de salud se capacitaron para que recibas una mejor atención? | Did you know that doctors at health centers have been trained to offer you better care? |
| 16 | Diabetes care | Algunas personas que tienen DIABETES pueden sentirse tristes o abatidas por la enfermedad. Si te sentís así háblalo con tu familia y con tu médico. | Some people who have DIABETES can feel sad or down about the disease. If you feel this way, talk with your family and your doctor. |
| 17 | Diabetes care | Si te medís el azúcar en sangre en tu casa con tiras, anota los valores y llévaselos al médico. Él va a darte o ajustarte el tratamiento. | If you measure your blood sugar at home with strips, take note of the values and give them to your doctor. They will give you or adjust your treatment. |
| 18 | Diabetes care | ¿Ya realizaste el taller de DIABETES? Recuerda que si aprendes a cuidarse, el azúcar en sangre va a bajar y vas a estar mejor! Acércate al centro de salud. | Have you already taken the DIABETES workshop? Remember that if you learn to take care of yourself, your blood sugar will go down and you will be better! Come to the health center. |
| 19 | Diabetes care | ¿Sabías que tener alta el azúcar en la sangre puede afectar la vista? Visita al oculista para chequear cómo están tus ojos. | Did you know that high blood sugar can affect your vision? Visit your optometrist to check how your eyes are. |
| 20 | Diabetes care | No te dejes estar. Si tenés DIABETES visita al médico regularmente para cuidarte y estar bien. Pedi un turno en el centro de salud. | Don’t put it off. If you have DIABETES, visit your doctor regularly to take care of yourself and stay healthy. Make an appointment at your health center. |
| 21 | Diabetes care | Para que la DIABETES esté bien controlada tenés que tener un valor de hemoglobina glicosilada de alrededor de 7. ¿Conoces cuál es tu valor de hemoglobina glicosilada ? | For DIABETES to be controlled well, you must have a glycosylated hemoglobin value of  around 7. Do you know your glycosylated hemoglobin value? |
| 22 | Follow-up | Si tenés DIABETES o azúcar en sangre alta, es importante un control con tu médico cada 3 meses en su centro de salud. Así vas a estar bien. | If you have DIABETES or high blood sugar, it is important to have a check-up with your doctor every 3 months at your health center. This will help keep you healthy. |
| 23 | Follow-up | Si sos diabético tenés que hacerte un fondo de ojo todos los años. ¿Cuándo fue la última vez que visitaste al oculista? | If you are diabetic, you must have an eye exam every year. When was the last time you visited an eye doctor? |
| 24 | Follow-up | ¿Cuándo fue la última vez que viste al médico? Si pasaron más de 3 meses desde la última visita, pedí un turno. | When was the last time you visited the doctor? If it has been more than 3 months since the last visit, make an appointment. |
| 25 | Follow-up | En la visita con el médico, habla los temas que son importantes para estar bien. (ej: qué análisis tenés que hacerte, mostrale los controles del azúcar en sangre o cómo aplicarte la insulina) | At the doctor’s appointment, talk about important issues to be healthy (ex. what tests you need to have, your blood sugar controls, or how to take your insulin). |
| 26 | Follow-up | Recuerda visitar a su médico al menos cada 3 meses para chequear cómo está tu DIABETES. | Remember to visit your doctor at least every 3 months to check in on your DIABETES. |
| 27 | Follow-up | ¿Sabías que para controlar el azúcar alta hay que hacerse un análisis en sangre de hemoglobina glicosilada cada 6 meses? Consulta al médico. | Did you know that to control high blood sugar, you should have a glycosylated hemoglobin blood test every 6 months? Consult your doctor. |
| 28 | Follow-up | Si pasaron más de 3 meses de la última vez que viste al médico, pedí a un turno. | If it has been more than 3 months since your last visit to the doctor, make an appointment. |
| 29 | Follow-up | Aunque te sientas bien, si tenés DIABETES o estás recibiendo un tratamiento para bajar el azúcar, visita al médico al menos cada 3 meses para un control. | Even if you feel fine, if you have DIABETES or are receiving treatment to lower your blood sugar, visit your doctor at least every 3 months for a check-up. |
| 30 | Follow-up | En la visita con el médico, habla los temas que son importantes para estar bien. (ej: qué análisis tenés que hacerte, cómo aplicarte la insulina o como cuidarte los pies). | At your doctor’s appointment, talk about the topics that are important for staying healthy (for example: which tests you need to do, how to take your insulin, or how to take care of your feet). |
| 31 | Follow-up | ¿Tenés controlados tus valores de azúcar en sangre? Consulta y controlate periódicamente con el médico de tu centro de salud. | Are you keeping your blood sugar levels under control? Visit your doctor regularly to check and manage them. |
| 32 | Follow-up | Aunque te sientas bien, visita al médico en tu centro de salud para un control. | Even if you feel fine, visit the doctor at your health center for a check-up. |
| 33 | Follow-up | ¿Sabías que para controlar el azúcar alta hay que hacerse un análisis en sangre de hemoglobina glicosilada cada 6 meses? Consulta al médico. | Did you know that to control high blood sugar you should have a glycosylated hemoglobin blood test every 6 months? Consult your doctor. |
| 34 | Follow-up | La única forma que tenemos de saber si nuestra presión arterial está bien, es tomándonos la presión. ¿Te tomaste la presión últimamente? | The only way we can know if our blood pressure is okay is to take our blood pressure. Have you taken your blood pressure lately? |
| 35 | Follow-up | Aunque te sientas bien, si tenés DIABETES o estás tomando medicamentos para el azúcar, visita a tu médico al menos cada 3 meses para un control | Even if you feel well, if you have DIABETES or are taking medication for blood sugar, visit your doctor at least every 3 months for a check-up. |
| 36 | Follow-up | En la visita con el médico, habla los temas que son importantes para estar bien. (ej: qué análisis tenes que hacerte o cómo tomar los medicamentos) | At the doctor's appointment, discuss the issues that are important for you to be well (e.g., what tests you need to have done or how to take your medications). |
| 37 | Foot care | ¿Te hiciste revisar los pies por un profesional de la salud? Tener azúcar alta en sangre puede afectar los nervios de las piernas de las personas con DIABETES. | Have you had your feet checked by a health professional? Having high blood sugar can affect the nerves in the legs of people with DIABETES. |
| 38 | Foot care | ¿Sabías que cuidar los pies es muy importante para las personas con DIABETES? Recuerda lavar y revisar sus pies todos los días. | Did you know that taking care of your feet is very important for people with DIABETES? |
| 39 | Foot care | Si tenés alguna lesión o lastimadura en el pie, consulta al médico de tu centro de salud. | If you have any injury or wound on your foot, consult the doctor at your health center. |
| 40 | Foot care | Evita caminar sin zapatos porque se pueden producir heridas en los pies. Las personas con DIABETES tienen los pies más sensibles. ¡Cuídelos! | Avoid walking without shoes because it can cause foot injuries. People with DIABETES have more sensitive feet; take care of them! |
| 41 | Foot care | Cuidar tus pies es muy importante. Lavalos todos los días con agua tibia, secalos bien y huméctalos con crema o aceite. | Taking care of your feet is very important. Wash them every day with warm water, dry them well, and moisturize with cream or oil. |
| 42 | Foot care | Para cuidar mejor tus pies, usa siempre medias de algodón. | To take better care of your feet, always wear cotton socks. |
| 43 | Lifestyle modification | Evita las gaseosas y los jugos artificiales porque aumentan el azúcar en sangre. Tu cuerpo te lo va a agradecer y te vas a sentir mejor. | Avoid sodas and artificial juices because they will increase your blood sugar. Your body will thank you, and you will feel better. |
| 44 | Lifestyle modification | La tentación de comer alimentos con grasa y dulces puede ser muy fuerte, pero recuerda que es importante estar bien. | The temptation to eat foods with fat and sugar can be very strong, but remember that it’s important to stay healthy and take care of your body. |
| 45 | Lifestyle modification | ¿Sabías que un plato saludable incluye hidratos de carbono (que están en el arroz o las lentejas), verduras y carnes? | Did you know that a healthy dish includes carbohydrates (such as rice or lentils), vegetables and meats? |
| 46 | Lifestyle modification | ¿Sabías que si bajas de peso vas a mejorar su salud? Te va a bajar el azúcar en la sangre, la presión arterial y también el colesterol. | Did you know that losing weight will improve your health? You will lower your blood sugar, blood pressure, and your cholesterol. |
| 47 | Lifestyle modification | ¿Estás estresado o preocupado? Haciendo actividad física 30 minutos al día vas a cuidar tu DIABETES y te vas a sentir más relajado y tranquilo. | Are you stressed or preoccupied? By doing physical activity for 30 minutes a day, you will take care of your DIABETES, and you will feel more relaxed and calmer. |
| 48 | Lifestyle modification | Recuerda que lo ideal es hacer 30 minutos al día de actividad física o ejercicio. Comienza con algo fácil y subí poco a poco la cantidad de tiempo. Comé algo antes de empezar. | Remember that the ideal is to do 30 minutes a day of physical activity or exercise. Start with something easy and gradually increase the amount of time. Eat something before you begin. |
| 49 | Lifestyle modification | ¿Sabías que los bizcochos, las galletitas, y las facturas suben el azúcar, la presión y el colesterol? | Did you know that biscuits, cookies, and pastries raise your blood sugar, blood pressure, and cholesterol? |
| 50 | Lifestyle modification | Para bajar la azúcar en sangre y no engordar, en lugar de gaseosas y jugos artificiales con azúcar, podés preparar una limonada casera con edulcorante . | To lower blood sugar and not gain weight, instead of sodas and sugary artificial juices, you can prepare homemade lemonade with sweetener. |
| 51 | Lifestyle modification | Cuida tu Diabetes comiendo frutas y verduras en la merienda en lugar de facturas, bizcochos, tortas y galletitas. | Take care of your diabetes by eating fruits and vegetables as a snack instead of pastries,  biscuits, cakes and cookies. |
| 52 | Lifestyle modification | Verduras como tomates, espinaca, brócolis y acelga ayudan a bajar de peso, bajar la azúcar y la presion y a mantenerse sano. | Vegetables such as tomatoes, spinach, broccoli, and chard help you lose weight, lower blood sugar and blood pressure, and stay healthy. |
| 53 | Lifestyle modification | Podés tener tu presión normal si usas menos sal en tu comida. Proteje tu corazón. | You can have normal blood pressure if you use less salt in your food. Protect your heart. |
| 54 | Lifestyle modification | Haciendo actividad física al menos 30 minutos al día va a bajar el azúcar, la presión y vas evitar enfermedades. No te olvides de hacer una colación antes (una fruta, un yogurt o una galleta con queso fresco) | Doing physical activity for at least 30 minutes a day will lower your blood sugar and blood pressure and prevent disease. Do not forget to have a snack before (a fruit, a yogurt, or a cracker with fresh cheese). |
| 55 | Lifestyle modification | ¿Sabías que podés comer sano y rico? ¿Cómo? Achicando las porciones y acompañando la alimentación con actividad física. | Did you know you can eat healthy and enjoy your food? How? By reducing portion sizes and combining your meals with physical activity. |
| 56 | Lifestyle modification | La tentación de comer alimentos con grasa y dulces puede ser muy fuerte, pero recuerda que es importante estar bien. | The temptation to eat fatty and sweet foods can be very strong, but remember that it’s important to stay healthy and take care of your body. |
| 57 | Lifestyle modification | Planifica hacer actividad física o el ejercicio que más te guste. Te vas a divertir y además te va a ayudar a bajar la presión, el colesterol y el azúcar. | Plan to do physical activity or exercise that you enjoy. You will have fun, and it will also help lower your blood pressure, cholesterol, and blood sugar. |
| 58 | Lifestyle modification | ¿Cuánto tiempo pasas mirando tele? ¡Para ser una persona activa sólo necesita 30 minutos al día de actividad física! | How much time do you spend watching TV? To be an active person, you only need 30 minutes a day of physical activity! |
| 59 | Lifestyle modification | Podés cuidar tu DIABETES. ¿Cómo? Haciendo ejercicio, evitando fumar, comiendo más verduras y frutas y eligiendo alimentos con menos azúcar y grasas. | You can take care of your DIABETES by exercising, avoiding smoking, eating more vegetables and fruits, and choosing foods with less sugar and fat. |
| 60 | Lifestyle modification | ¿Sabías que si bajas de peso vas a mejorar su salud? Te va a bajar el azúcar en la sangre, la presión arterial y también el colesterol. | Did you know that if you lose weight, your health will improve? It will lower your blood sugar, blood pressure, and cholesterol. |
| 61 | Lifestyle modification | En vez de ver tele, busca amigos o familiares que te acompañen a caminar o a hacer otra actividad física. Eso lo hará fácil y entretenido | Instead of watching TV, find friends or family members to join you for a walk or other physical activity. That will make it easy and fun. |
| 62 | Lifestyle modification | Haciendo actividad física al menos 30 minutos al día te va a bajar el azúcar, la presión y vas evitar enfermedades. | Doing physical activity at least 30 minutes a day will lower your blood sugar and blood pressure and prevent illnesses. |
| 63 | Lifestyle modification | No agregues azúcar al café, al mate o al té. Usar edulcorante te ayudará a controlar la DIABETES. | Do not add sugar to your coffee, mate, or tea. Using sweeteners will help you control DIABETES. |
| 64 | Medication adherence | Trata de no olvidarte de tomar los medicamentos y de aplicarse la insulina para la DIABETES porque si no el tratamiento no funciona. | Try not to forget to take your medications and take insulin for your DIABETES; otherwise, the treatment will not work. |
| 65 | Medication adherence | El tratamiento para la DIABETES dura toda la vida. Acostumbrarse a hacer el tratamiento todos los días te ayudará a que vivas más y mejor. | The treatment for DIABETES lasts a lifetime. Getting used to do the treatment every day will help you live longer and better. |
| 66 | Medication adherence | Si te bajan los valores del azúcar en sangre es porque el medicamento te hace efecto. ¡Por eso es importante seguirlo tomando siempre! | If your blood sugar levels go down, it is because the medication has taken effect. This is why it is important to continue taking it all the time! |
| 67 | Medication adherence | Si el médico te receta medicamentos para la DIABETES, tómalos siguiendo sus indicaciones. No los suspendas por tu cuenta, consulta siempre. | If your doctor prescribes medication for DIABETES, take it as directed. Do not stop taking them on your own, always consult your doctor. |
| 68 | Medication adherence | Si te sentís mal y pensás que es por los medicamentos que estás tomando, consulta con el médico. | If you feel sick and think it is because of the medications you are taking, consult your doctor. |
| 69 | Medication adherence | Trata de no olvidarte de tomar tus medicamentos para la DIABETES porque si no los tomás, el tratamiento no funciona. | Try not to forget to take your DIABETES medications because if you do not take them, the treatment does not work. |
| 70 | Medication adherence | ¿Sabías que los medicamentos para la DIABETES y la insulina se entregan gratis para las personas que no tienen obra social? | Did you know that DIABETES medications and insulin are provided free of charge for people who do not have social security? |
| 71 | Medication adherence | No dejes de tomar el medicamento para la DIABETES. Si lo abandonas, el azúcar en sangre volverá a subir. | Do not stop taking your DIABETES medicine. If you stop, your blood sugar will go back up. |
| 72 | Medication adherence | Fíjate cuántos medicamentos para la DIABETES le quedan. Visita al médico para conseguir una receta antes de que se te acaben. | See how much DIABETES medication you have left. Visit your doctor to get a prescription before you run out. |
| 73 | Medication adherence | Los medicamentos para la DIABETES funcionan muy bien y bajan el azúcar en sangre cuando los tomás como le indicó el médico. | DIABETES medications work very well and lower blood sugar when you take them as prescribed by your doctor. |
| 74 | Medication adherence | Los medicamentos que te da el médico, para la DIABETES, la presión y el colesterol, ayudan a que tu corazón esté bien. | The medicines your doctor gives you for DIABETES, blood pressure, and cholesterol help your heart to be well. |
| 75 | Medication adherence | Si el médico te receta medicamentos para la DIABETES, tómalos siguiendo sus indicaciones. No los suspendas por tu cuenta, consulta siempre. | If the doctor prescribes medication for DIABETES, take them according to the instructions. Do not stop taking them on your own, always consult your doctor. |
| 76 | Medication adherence | Fíjate cuántos medicamentos para la DIABETES le quedan. Visita al médico para conseguir una receta antes de que se te acaben | See how much DIABETES medication you have left. Visit your doctor to get a prescription before you run out. |
| 77 | Insulin use | Recuerda cambiar el lugar del cuerpo donde te aplica la insulina. Podés aplicarte en un mismo lugar durante una semana y después cambiar a otro. | Remember to change the spot on your body where you inject your insulin. You can use the same area for a week and then switch to another one. |
| 78 | Insulin use | La insulina evita que la DIABETES avance. Ayuda a que el azúcar en la sangre no suba. | The insulin you take stops the DIABETES from progressing. It helps keep your blood sugar from rising. |
| 79 | Insulin use | Si notas que en el lugar donde te inyecta la insulina tenes como pelotitas duras, avisale al médico. | If you notice that the place where you inject insulin has a hard ball-like appearance, tell the doctor. |
| 80 | Insulin use | Si usas insulina, come una fruta, 1/4 de pan o 3 galletitas de agua antes y después de realizar algún ejercicio físico. Así evitarás que te baje mucho el azúcar. | If you take insulin, eat one fruit, ¼ piece of bread, or 3 water cookies before and after you exercise. This should keep your blood sugar from dropping too low. |
| 81 | Insulin use | No dejes de aplicarte la insulina para la DIABETES. Si abandonas el tratamiento, el azúcar en sangre volverá a subir y no te vas a sentir bien. | Do not stop taking insulin for DIABETES. If you abandon the treatment, your blood sugar will rise again, and you will not feel well. |
| 82 | Insulin use | Fijate cuántas lapiceras con insulina te quedan. Visita al médico para conseguir una receta antes de que se te acaben. | See how many insulin pens you have left. Visit the doctor to continue your prescription before you run out. |
| 83 | Insulin use | No dejes la lapicera de insulina que estás usando expuesta al calor o la luz. Cuando hace mucho calor, después de la aplicación, guardala en la puerta de la heladera. | Do not leave the insulin pen you are using exposed to heat or light. In very hot weather, store it in the fridge door after application. |
| 84 | Insulin use | Si notas que en el lugar donde te inyecta la insulina tenés como pelotitas duras, avisale al médico | If you notice hard lumps where you inject your insulin, let your doctor know. |
| 85 | Hypoglycemia prevention | Recuerda que para evitar tener un bajón de azúcar (debajo de 70) es importante que no saltees comidas ni hagas ayunos prolongados. | Did you know that when your blood sugar is high, you may feel dizzy, have blurry vision, feel tired, and urinate more? If this happens to you, consult your doctor. |
| 86 | Hypoglycemia prevention | Si tenes DIABETES y tomas medicamentos o te aplicas insulina, ¡nunca tomes alcohol con el estómago vacío! Puede bajarte mucho el azúcar y sentirte mal. | If you have DIABETES and take medication or insulin, never drink alcohol on an empty stomach! It can make your blood sugar very low and make you feel sick. |
| 87 | Hypoglycemia prevention | Si tomás medicamentos para la DIABETES o recibís insulina, ¡no ayunes ni saltees las comidas! Así evitarás hipoglucemias. | If you take DIABETES medication or insulin, do not fast or skip meals! This way you will avoid hypoglycemia. |
| 88 | Hypoglycemia prevention | Si te baja el azúcar y tenes dolor de cabeza, transpiras frío, o te mareas, come 5 caramelos o toma un vaso de agua con tres cucharadas de azúcar. | If your blood sugar is low and you have a headache, sweat, or feel dizzy, eat five pieces of candy or drink a glass of water with three tablespoons of sugar. |

**For people with moderate or high risk of developing diabetes**

| **Number** | **Dimensions** | **Spanish version of the SMS** | **English translation** |
| --- | --- | --- | --- |
| 1 | Introduction | ¿Sabías que la DIABETES es una enfermedad que se puede prevenir? | Did you know that DIABETES is a disease that can be prevented? |
| 2 | Introduction | ¡Perder peso y realizar actividad física puede ayudarnos a prevenir la diabetes! | Losing weight and engaging in physical activity can help us prevent diabetes! |
| 3 | Introduction | La PREDIABETES es un estado anterior a la diabetes, en donde el azúcar en sangre (glucemia) está en valores por encima de lo normal (entre 110 y 125 mg/dl) | PREDIABETES is a stage before diabetes, where blood sugar levels (glycemia) are higher than normal (between 110 and 125 mg/dl). |
| 4 | Introduction | La prediabetes se puede corregir y con esto, evitar la aparición de la diabetes. Para ello podemos revisar nuestros hábitos y buscar las opciones más saludables. | Prediabetes can be reversed, helping to prevent the onset of diabetes. To achieve this, we can evaluate our habits and choose healthier options. |
| 5 | Introduction | Las metas son algo que deseamos, planeamos y nos comprometemos a hacer. Es importante pensar nuestras metas antes de pensar un cambio de hábito. ¿Pensaste alguna vez en qué metas querés cumplir? | Goals are something we desire, plan for, and commit to achieving. It’s important to consider our goals before considering a change in habits. Have you ever thought about what goals you want to accomplish? |
| 6 | Introduction | La insulina evita que la DIABETES avance. Ayuda a que el azúcar en la sangre no suba. | Insulin prevents diabetes from progressing. It helps keep blood sugar levels from rising. |
| 7 | Introduction | Recordá siempre: todo cambio de hábito, por pequeño que parezca, ¡es MUY IMPORTANTE! | Always remember: every change of habit, no matter how small it may seem, is VERY IMPORTANT! |
| 8 | Introduction | CUIDADO con el "Efecto Rebote". Si bajás mucho de peso y después volvés a los viejos hábitos, vas a subir de manera muy rápida de peso. | BE CAREFUL with the “Rebound Effect.” If you lose weight and then return to your old habits, you will gain weight quickly. |
| 9 | Physical activity | Lo ideal es hacer 30 minutos de actividad física todos los días. Podés empezar con unos pocos minutos y después ir subiendo poco a poco la cantidad de tiempo. | Ideally, you should aim for 30 minutes of physical activity every day. You can start with just a few minutes and gradually increase the duration. |
| 10 | Physical activity | Hacer actividad física debe ser algo que nos guste, al aumentar la actividad física nos sentimos mejor y nos ayuda a bajar el azúcar en sangre. | Exercise should be something you enjoy. Increasing physical activity makes you feel better and helps lower blood sugar levels. |
| 11 | Physical activity | Hay 1440 minutos en el día. ¿Cuántos minutos usted PUEDE DEDICAR para realizar Actividad Física en el día? | There are 1,440 minutes in a day. How many minutes can you dedicate to physical activity today? |
| 12 | Physical activity | ¡Limpiar la casa, subir escaleras, trabajar en el jardín, son actividades que nos ayudan a movernos y a bajar el azúcar en sangre! | Cleaning the house, climbing stairs, and working in the garden, are activities that help us move and lower our blood sugar! |
| 13 | Physical activity | ¿Llegás cansado y no te dan ganas de hacer actividad física? Ir al trabajo en bicicleta, hacer las compras o llevar a los chicos caminando puede ayudarte a sentirte mejor. | Are you tired and don't feel like doing physical activity? Biking to work, shopping, or walking with your kids can help you feel better. |
| 14 | Physical activity | ¿Querés empezar a hacer actividad física? Buscá algo que te divierta. ¿Qué te parece bailar? Lo podés hacer solo/a o acompañado. Es una muy buena decisión, ¡VAMOS! | Want to get active? Find something fun to do. How about dancing? You can do it alone or with company. It's a very good decision, LET'S GO! |
| 15 | Physical activity | Hacer actividad física a veces puede ser difícil. Es más fácil si buscamos un amigo o un familiar que nos acompañe, buscá la actividad que más te guste. | Doing physical activity can sometimes be difficult. It is easier if we look for a friend or a family member to accompany us; find the activity you enjoy the most. |
| 16 | Physical activity | Hacer las compras caminando o en bici, hacer las tareas de la casa o pasear a tu mascota te ayudan a aumentar tu actividad física! | Shopping, walking or biking, doing housework, or walking your pet will help you increase your physical activity! |
| 17 | Physical activity | Dividamos el tiempo para hacer actividad física: 20 minutos todos los días o 50 minutos 3 veces a la semana. ¡Buscá la que sea más cómoda y no te olvides de divertirte! | Let's split up the time for physical activity: 20 minutes every day or 50 minutes 3 times a week, whichever is more comfortable and don't forget to have fun! |
| 18 | Physical activity | Para que te sea más fácil hacer actividad física, buscá una actividad que te dé placer, te divierta y te guste. | To make it easier for you to do physical activity, find an activity that gives you pleasure, is fun, and that you enjoy. |
| 19 | Physical activity | ¿Por qué anotar la Actividad Física que hacemos? Anotar es un apoyo para darse cuenta de cómo va mejorando la condición física. | Why write down the physical activity we do? Recording is a way to keep track of how your physical condition is improving. |
| 20 | Physical activity | Anotar la Actividad Física que hacés ayuda para ir aumentando poco a poco la cantidad de ejercicio que hacemos para llegar a nuestra meta. ¡Ánimo! | Writing down the physical activity you do helps you gradually increase the amount of exercise you get, so you can reach your goal. Keep it up! |
| 21 | Physical activity | Para llevar un registro de la actividad física, podemos usar un cronómetro o un reloj. Hay muchas formas de ver cuánto ejercicio hacemos. | We can use a stopwatch or a watch to keep track of physical activity. There are many ways to see how much exercise we do. |
| 22 | Physical activity | Sabemos que hacer ejercicio puede ser difícil y, aunque ya vengas haciendo ejercicio, puede que un día no quieras ir, pero vamos ¡no pierdas las ganas! | We know that exercising can be difficult, and even if you are already exercising, you may not want to go one day, but come on, don't lose the motivation! |
| 23 | Healthy diet | ¿Sabías que si cambiás gaseosa o agua saborizada por agua o bebidas dietéticas, bajás de peso y evitás enfermedades? | Did you know that by switching from soda or flavored drinks to water or diet beverages, you can lose weight and prevent diseases? |
| 24 | Healthy diet | ¿Te sentís triste o estresado y te dan ganas de comer chocolate o cosas dulces? Esto se le llama "hambre emocional" y es importante reconocer si nos está pasando. | Do you feel sad or stressed and crave chocolate or sweets? This is called 'emotional hunger,' and it's important to recognize when it's happening. |
| 25 | Healthy diet | El hambre emocional tiene que ver con nuestro estado de ánimo y muchas veces nos lleva a comer de más. Probá hablar con alguien de lo que te pasa o escribilo en un papel. Esto puede servir como pausa antes de la comida. | Emotional hunger is linked to our mood and often leads us to overeat. Try talking to someone about how you feel or writing it down on paper. This can help you take a pause before you eat . |
| 26 | Healthy diet | ¿Durante el día comés un pedacito de queso o una galletita fuera de hora? Mientras cocinas, ¿comés un poquito de lo que haya? ¡Eso se llama picoteo y puede hacernos subir de peso! | During the day, do you eat a piece of cheese or a cracker after hours? While you are cooking, do you eat a little bit of whatever is available? This is called snacking and can make us gain weight! |
| 27 | Healthy diet | Si estás mal, triste o aburrido, evitá comer cosas dulces. ¡En esos momentos es mejor salir a caminar con un amigo o familiar, hablar de nuestros sentimientos o hacer cosas que nos gusten! | Avoid eating sweet things if you feel bad, sad, or bored. In those moments it is better to go for a walk with a friend or family member, talk about our feelings, or do things you enjoy! |
| 28 | Healthy diet | ¿Cómo evitar tentaciones si querés bajar de peso? Guardá los alimentos menos saludables (como chocolate, galletitas dulces, papas fritas, etc.) en lugares que no estén al alcance ayuda a evitar tentaciones. | How to avoid temptations if you want to lose weight? Keep less healthy foods (such as chocolate, cookies, chips, etc.) out of reach . |
| 29 | Healthy diet | ¿Qué es un snack saludable? Son alimentos que podemos usar para "picar" que tienen pocas calorías. Por ejemplo, bastones de zanahoria o chips de verduras al horno (berenjena, calabaza, pepino). Son fáciles de preparar y muy ricos. | What is a healthy snack? They are foods that we can use for “snacking” that are low in calories. For example, carrot sticks or baked vegetable chips (eggplant, pumpkin, cucumber). They are easy to prepare and very tasty. |
| 30 | Healthy diet | ¿Es difícil dejar el "picoteo"? Una buena opción para no subir de peso es tener a mano "picoteos saludables", como por ejemplo: chips de frutas o verduras desecadas, bastoncitos de apio o zanahoria, semillas de clabaza tostadas. | Is it hard to give up “snacking”? A good way to keep the weight off is to have “healthy snacks” on hand, such as dried fruit chips or vegetable chips, celery or carrot sticks, and roasted pumpkin seeds. |
| 31 | Healthy diet | ¿Sabías que las lentejas y los porotos son alimentos con mucha fibra que te va a ayudar a sentirte lleno? Estos alimentos nos ayudan a evitar el picoteo y pueden combinarse con muchos otros alimentos. | Did you know that lentils and beans are high fiber foods that will help you feel full? These foods help us avoid snacking and can be combined with many other foods. |
| 32 | Healthy diet | ¿Sabés que anotar lo que comemos nos ayuda a saber qué y cuánto estamos comiendo? Esto ayuda a controlar nuestro peso. Podés dejar un anotador en la cocina para no olvidarte de escribir lo que comés. | Did you know that writing down what we eat helps us know what and how much we eat? This helps to control our weight. You can keep a notebook in the kitchen so you don't forget to write down what you eat. |
| 33 | Healthy diet | Si preparás tus comidas al horno, a la plancha o al vapor, podés evitar el consumo de grasas agregadas por cocción y eso te ayuda a bajar de peso. Evitá fritos y salteados. | If you cook your meals by baking, grilling, or steaming them, you can avoid extra fats from cooking, which helps with weight loss. Try to avoid fried and sautéed foods. |
| 34 | Healthy diet | Los cumpleaños o reuniones familiares pueden llevarnos a comer de más. Preparar snacks saludables para compartir. Cambiar las papas fritas por chips de vegetales al horno, brochetes de vegetales con aderezos de espinaca o zanahora, son algunas opciones ricas y saludables para compartir. | Birthdays or family gatherings can lead us to overeat. Prepare healthy snacks to share. Swap french fries for baked vegetable chips or vegetable skewers with spinach or carrot toppings are some tasty and healthy options to share. |
| 35 | Healthy diet | Si tengo hambre, puedo comer un snack saludable antes de salir de casa (una fruta, algunas frutas secas, turrón, etc.) | If I'm hungry, I can eat a healthy snack before I leave the house (a piece of fruit, some dried fruit, nougat, etc.) |
| 36 | Healthy diet | CUIDADO: ¡Ir a hacer las compras con hambre nos puede llevar a tentarnos con alimentos poco saludables! Comé algo antes de ir al mercado o esperá otro momento para no tentarte. | BEWARE: Shopping hungry can lead to temptation with unhealthy foods! Eat something before going to the market or wait until another time to avoid being tempted. |
| 37 | Healthy diet | Cuando vayas al supermercado, ¡cuidado con los envases o paquetes grandes de alimentos no saludables! ¡Suelen ser más baratos y terminamos comiendo de más! | When you go to the supermarket, watch out for large packages or packages of unhealthy foods! They are usually cheaper, and we end up overeating! |
| 38 | Healthy diet | Recuerda que para evitar tener un bajón de azúcar (debajo de 70) es importante que no saltees comidas ni hagas ayunos prolongados. | Remember that to avoid having a sugar crash (below 70), it is important not to skip meals or fast for long periods of time. |
| 39 | Healthy diet | ¿Probaste endulzar el mate, el té o el café con edulcorante? ¡Hay muchos tipos de edulcorante, si uno no te gusta podés probar con otros! Evitá ponerle azúcar. | Have you tried sweetening your mate, tea, or coffee with sweetener? There are many sweeteners; if you don't like one, you can try others! Avoid adding sugar. |
| 40 | Healthy diet | Reducir la cantidad de azúcar que le agregás al café, al mate o al té te ayudará a bajar de peso y a controlar el azúcar en sangre. | Reducing the amount of sugar you add to coffee, mate, or tea will help you lose weight and control your blood sugar. |
| 41 | Healthy diet | ¿Sabías que la piel del pollo tiene mucha grasa? Sacarla antes de cocinar te ayuda a comer menos calorías y a cuidar tu salud y la de tu familia | Did you know that chicken skin has a lot of fat? Removing it before cooking helps you eat fewer calories and take care of your health and the health of your family. |
| 42 | Healthy diet | Tips para evitar el picoteo no saludable: respetá las 4 comidas (desayuno, almuerzo, merienda y cena). Si te quedás con hambre, planificá colaciones saludables. Tomá mucha agua, comé alimentos con mucha fibra como frutas y verduras. | Tips to avoid unhealthy snacking: respect the four meals (breakfast, lunch, afternoon snack, and dinner). If you get hungry, plan healthy snacks. Drink plenty of water, and eat foods with lots of fiber, such as fruits and vegetables. |
| 43 | Healthy diet | Disminuir el consumo de gaseosas y jugos artificiales (como Carioca, Mocoretá, Tang) te va a ayudar a bajar de peso y a controlar el azúcar en sangre. ¿Qué te parece solo tomarlo en ocasiones especiales? | Decreasing the consumption of sodas and artificial juices will help you lose weight and control your blood sugar. How about drinking these only on special occasions? |
| 44 | Healthy diet | El consumo de agua nos ayuda a mantener las funciones del cuerpo y nos ayuda a bajar de peso. | Water consumption helps us to maintain our body functions and helps us to lose weight. |
| 45 | Healthy diet | ¿Queres tomar más agua? Podés llevar siempre una botella con vos. | Want to drink more water? You can always carry a bottle with you. |
| 46 | Healthy diet | ¿Sabías que la grasa que vemos en la carne tiene muchas calorías? Sacar toda la grasa visible de las carnes antes de cocinarla nos ayuda a cuidar nuestra salud. | Did you know that the fat we see in meat has a lot of calories? Removing all the visible fat from meats before cooking helps us take care of our health. |
| 47 | Healthy diet | ¿Te pasó de ir a una fiesta y comer de más? Es muy fácil perder la noción de lo que uno come en estos eventos. Para que esto no pase, servirte en un plato todo lo que decidas comer al inicio de la reunión y evitá repetir platos. | Have you ever gone to a party and overeate? It is easy to lose track of what you eat at these events. So that this does not happen, serve yourself everything you decide to eat at the beginning of the event and avoid repeating dishes. |
| 48 | Healthy diet | ¿Sabías que las semillas de calabaza tostadas se pueden comer? Tienen mucha fibra que nos hacen sentir más llenos, calman el hambre y reducen el "picoteo". También las podés conseguir en el supermercado ya preparadas! | Did you know that roasted pumpkin seeds can be eaten? They have a lot of fiber which makes us feel fuller, reduces hunger, and decreases “snacking.” You can get them in the supermarket already prepared! |
| 49 | Healthy diet | ¿Cómo usamos las semillas? Las semillas de gisasol son muy saludables y podemos tostarlas y utilizarlas snack saludable. Tené cuidado con la cantidad, solo un puñado! | How do we use seeds? Sunflower seeds are very healthy; we can toast them and use them as a healthy snack. Be careful with the quantity, just a handful! |
| 50 | Healthy diet | Es muy fácil comer de más cuando tu atención está en otra cosa. Si te agarra hambre cuando estás mirando la tele, podés poner una pequeña cantidad de frutas o cereales sin azúcar en un plato. | It is very easy to overeat when your attention is on something else. If you get hungry when you're watching TV, consider putting a small amount of fruit or unsweetened cereal on a plate. |
| 51 | Healthy diet | No comás directo de la bolsa, poné la cantidad que quieras comer en un plato pequeño, así evitás comer en exceso! | Don't eat anything straight from the bag; put the amount you want to eat on a small plate so you avoid overeating! |
| 52 | Healthy diet | ¿Qué te parece si antes de salir de casa hacés una lista de los alimentos que necesitás? De esta manera, ahorrás dinero y evitás las tentaciones de comprar alimentos no saludables. | What do you think about making a list of the foods you need to buy at the grocery store before leaving home? This way, you will save money and avoid the temptation to buy unhealthy foods. |
| 53 | Healthy diet | ¿Sabías que si antes del "picoteo" tomás un vaso de agua y esperás unos minutos, disminuyen las ganas de comer? | Did you know that if you drink a glass of water and wait a few minutes before “snacking”, the urge to eat will decrease? |
| 54 | Healthy diet | No hay ningún alimento prohibido. ¡En fiestas o cumpleaños no te prives de comer ni tomar nada que te guste! Pero OJO, ¡¡cuidá las cantidades!!! | There is no forbidden food. Do not deprive yourself of eating or drinking anything you like at parties or birthdays! Just be careful with the quantities. |
| 55 | Healthy diet | Un tropezón no es una caída. Si hoy comiste de más no te preocupes, siempre se puede volver a empezar. | A stumble is not a fall. If today you overate, don't worry, you can always start again. |
| 56 | Motivation | ¿Qué podés hacer hoy para acercarte a cumplir el cambio de hábito que deseas? Es importante ir cambiando poco a poco. Un pequeño cambio es de gran ayuda. | What can you do today to move closer to achieving the habit change you desire? It’s important to make gradual changes. A small change can make a big difference. |
| 57 | Motivation | Escribí tus logros o compartilos con alguien que quieras. Poco a poco notarás cómo todo pequeño cambio suma. | Write down your achievements or share them with someone you love. Little by little, you will notice how every little change adds up. |
| 58 | Motivation | ¡No es necesario prohibir ningún alimento! Podemos controlar la frecuencia (la cantidad de veces) con la que comemos alimentos como: facturas, fiambres y gaseosas. | It is not necessary to prohibit any food! We can control the frequency (the amount of times) that we eat foods such as: pastries , cold cuts and soda. |
| 59 | Behavior change | Para hacer un cambio de hábito, es importante desearlo, pensar cómo hacerlo (planificarlo) y comprometernos con ese deseo. Una buena manera de planificar es escribir las metas y cada paso que tenés que dar para lograrlo. | To change a habit, it’s important to truly want it, to think about how to make it happen (plan it), and to commit to that goal. A good way to plan is by writing down your goals and each step you need to take to achieve them. |
| 60 | Behavior change | ¡No es necesario cambiar todo de un día para otro! Los pequeños cambios nos ayudan a ir mejorando poco a poco y a sentirnos mejor. | It is not necessary to change everything in one day! Small changes help us to improve little by little and feel better. |
| 61 | Behavior change | Compartí tus planes de cambio de hábitos con alguien que quieras. Ellos pueden ayudarte a cumplir tus metas | Share your habit change plans with someone you love. They can help you meet your goals. |
| 62 | Behavior change | Cada cambio en tus hábitos (comidas o actividad física) que hagas, por mas pequeño que creas que sea, es MUY importante para tu salud! | Every change in your habits (food or physical activity) you make, no matter how small you think it is, is significant for your health! |
| 63 | Weight control | ¿Tenés que bajar de peso y no sabés cómo? Empezá de a poco: disminuir las porciones de cada comida, o comer más frutas y verduras te va a ayudar a bajar de peso. | Do you need to lose weight and don’t know how? Start small: reducing portion sizes or eating more fruits and vegetables will help you lose weight. |
| 64 | Weight control | Si querés bajar de peso, es importante que sea de a poco, para que se mantenga a lo largo del tiempo. Esto nos ayuda a evitar el "Efecto Rebote" o volver a subir rápido. | If you want to lose weight, it's important to do it gradually so that the results last over time. This helps prevent the 'Rebound Effect' or quickly regaining the lost weight. |
| 65 | Weight control | Perder peso puede tener muchos beneficios: previene la diabetes, disminuye la presión arterial, y el colesterol y te ayuda a dormir mejor! | Losing weight has many benefits: it helps prevent diabetes, lowers blood pressure and cholesterol, and improves sleep quality! |

**For pregnant women**

| **Number** | **Spanish version of the SMS** | **English translation** |
| --- | --- | --- |
| **Welcome** | ¡Hola! Te damos la bienvenida al programa de prevención de la diabetes en el embarazo. Son muy importantes los cuidados y controles durante el embarazo, tanto si ya fuiste mamá como si esperás a tu primer bebé. Todas las semanas te vamos a acompañar con mensajes con información importante sobre alimentacion saludable y cuidados para vos y tu bebé que pueden ayudarte a prevenir la diabetes del embarazo. Ante cualquier duda o consulta, podés concurrir al centro de salud donde realices los controles de tu embarazo. | Hello! Welcome to the gestational diabetes prevention program. Proper care and regular check-ups during pregnancy are very important, whether you have been a mom before or are expecting your first baby. Every week, we will send you messages containing important information about healthy eating habits and care for you and your baby that can help you prevent gestational diabetes. If you have any questions or concerns, visit the health center where you have your prenatal check-ups and they will answer any questions you have. |
| **1** | ¿Sabías que... los antojos no satisfechos no dejan marcas de nacimiento en tu bebé? Es normal tener antojos durante el embarazo, pero no dejan marcas en tu bebé. Ciertas marcas en la piel del recién nacido se consideran normales | Did you know that unsatisfied cravings do not leave birthmarks on your baby? It’s normal to have cravings during pregnancy, and they don’t leave marks on your baby. Certain marks on a newborn’s skin are considered normal. |
| **2** | ¿Tenés acidez? Algunos alimentos que te pueden ayudar durante el embarazo son la leche fría, frutas cocidas o en compota sin la cáscara. Evitá las golosinas o confituras, las facturas, los alimentos fritos y el café. Eso te hará sentir mejor! | Do you suffer from heartburn? Some foods that can help relieve this during pregnancy are cold milk, cooked fruits, or fruit jam without the skin. Avoid sweets or candies, pastries, fried foods, and coffee. That will make you feel better! |
| **3** | En los Centros de Atención Primaria de la Salud (CAPS o "salitas") hay profesionales de la salud que pueden controlar tu embarazo: una obstétrica, un médico obstetra o un médico de familia. | At the Primary Health Care Centers (PHCC), there are health professionals who can monitor your pregnancy: a midwife, an obstetrician, or a family doctor. |
| **4** | Al comenzar las consultas en el hospital o en los CAPS, te van a entregar un Carné Perinatal. Es importante que lo lleves a cada control. Tiene información muy importante y necesaria para atenderte. | At the beginning of your first appointment at the PHCC, you will be given a Perinatal Card. It is important to bring it to every check-up because it contains very important and necessary information for your care. |
| **5** | Lo ideal es que durante los primeros 7 meses de emabarazo te hagas un control mensual, luego cada 15 días y en el último mes te controles todas las semanas. | Ideally, during the first 7 months of pregnancy you should have a monthly check-up, then every 15 days, and in the last month, you should be monitored every week. |
| **6** | Si fumás, el embarazo es un buen momento para intentar dejarlo, va a mejorar tu salud y la de tu bebé. Sabemos que es una decisión muy difícil, pero es posible! Comentalo en tus controles médicos, el equipo de salud puede ayudarte y acompañarte en este camino. Animate! | If you smoke, pregnancy is a great time to try quitting—it will improve both your health and your baby’s. We know it’s a very difficult decision, but it’s possible! Discuss it during your medical check-ups; the health team can help and support you on this journey. You can do it! |
| **7** | Por más que tengas una alimentación saludable, el equipo de salud te indicará tomar ácido fólico durante los primeros tres meses. Esto es muy importante para la salud de tu bebé, ya que ayuda a que el bebé crezca sano. | Even if you maintain a healthy diet, the health team will advise you to take folic acid during the first three months. This is very important for your baby’s health, as it helps your baby grow healthily. |
| **8** | ¿Sabías que la diabetes gestacional o diabetes del embarazo aparece cuando los niveles de azúcar en sangre son más altos de lo normal? Puede generar complicaciones en la mamá y el bebé. ¡Por eso es importante que siempre realices los controles y análisis que te indique el equipo de salud! | Did you know that gestational diabetes—or pregnancy diabetes—occurs when blood sugar levels are higher than normal? It can cause complications for both the mother and the baby. That’s why it’s important to always attend the check-ups and tests recommended by your healthcare team! |
| **9** | Ahora que estoy embarazada, ¿hay algunos alimentos que no pueda comer? ¡No, no existen alimentos prohibidos! Sí debemos fijarnos en la cantidad que comemos y el tamaño de las porciones. ¡También debemos asegurarnos de que las carnes estén bien cocidas, que las verduras estén bien lavadas y que el agua que tomemos sea segura para el consumo! | Now that I’m pregnant, are there any foods I can’t eat? No, there are no forbidden foods! We just need to pay attention to the amount we eat and the size of our portions. We also need to ensure that meats are well-cooked, vegetables are thoroughly washed, and that the water we drink is safe to drink! |
| **10** | ¿Querés comer más sano pero no estás segura de cómo hacerlo? Consultá en el centro de salud (salita) u hospital más cercano! Podés empezar incorporando mayor cantidad de frutas y verduras de las que más te gusten. Aprovechá las frutas y verduras de estación que son más baratas! | Do you want to eat healthier but aren’t sure how to do it? Check with the nearest health center (PHCC) or hospital! You can start by incorporating more of your favorite fruits and vegetables. Take advantage of seasonal fruits and vegetables, which tend to be less expensive! |
| **11** | Hacer ejercicio durante el embarazo es muy bueno. Las caminatas, a tu ritmo, sin cansarte demasiado, son un excelente ejercicio. Consultá con el profesional de salud que atiende tu embarazo qué tipo de ejercicio es bueno para tu salud y la de tu bebé. | Exercising during pregnancy is very beneficial. Walking at your own pace, without overexerting yourself, is an excellent form of exercise. Consult with the healthcare professional managing your pregnancy about which type of exercise is best for you and your baby. |
| **12** | La actividad física realizada regularmente durante el embarazo puede mejorar tu estado de ánimo y ayudarte a dormir mejor! | Regular physical activity during pregnancy can improve your mood and help you sleep better! |
| **13** | Cuando estás embarazada, las necesidades de hierro aumentan para cubrir tus necesidades y las de tu bebé. El hierro es importante para el desarrollo físico y neurológico del bebé y para evitar la anemia durante el embarazo. | When you are pregnant, your body's need for iron increases to support both your own health and your baby's development. Iron is essential for your baby’s physical and neurological development and for preventing anemia during pregnancy. |
| **14** | La carne, las lentejas, nueces, brócoli o espinaca son alimentos ricos en hierro. Dato: para ayudar a la absorción del hierro, es bueno tomar un jugo de naranja después de comer estos alimentos o condimentarlos con jugo de limón! | Meat, lentils, nuts, broccoli, or spinach are all foods rich in iron. Tip: To aid iron absorption, it’s helpful to drink a glass of orange juice after eating these foods or to season them with lemon juice! |
| **15** | Algunas mujeres tienen sobrepeso cuando quedan embarazadas y otras aumentan de peso demasiado rápido durante su embarazo. En estos casos se recomienda no hacer ninguna dieta ni bajar de peso. Es mejor enfocarse en una alimentación saludable y mantenerse activa | Some women are overweight when they become pregnant, and others gain weight too quickly during their pregnancy. In these cases, it is recommended not to go on any diet or try to lose weight. It’s better to focus on healthy eating and staying active. |
| **16** | ¿Ya fuiste al control? No te olvides siempre de llevar el Carné Perinatal para que el equipo de salud anote los resultados de cada control. | Have you already attended your check-up? Don’t forget to always bring your Perinatal Card so that the healthcare team can record the results of each visit! |
| **17** | Es importante que asistas a todos los controles del embarazo. ¡Si no pudiste ir a uno de los turnos, no olvides sacar otro! El equipo de salud puede ayudarte con esto. | It is important that you attend all your pregnancy check-ups. If you miss one appointment, be sure to schedule another! The healthcare team can help you with this. |
| **18** | ¿Sabías que durante el embarazo tenés que vacunarte contra la gripe, "coqueluche" y el COVID? La vacuna contra la gripe y la vacuna contra el COVID te las podés dar en cualquier trimestre, la triple bacteriana acelular contra el coqueluche partir de la semana 20. Averiguá con el equipo de salud que te atiende dónde podés vacunarte. | Did you know that during pregnancy you need to be vaccinated against the flu, whooping cough, and COVID? The flu vaccine and the COVID vaccine can be administered in any trimester, while the acellular pertussis triple vaccine for whooping cough should be given after week 20. Check with your healthcare team where you can get vaccinated. |
| **19** | COMIDA RICA Y SALUDABLE. Si tenés hambre entre comidas, podés prepararte cosas saludables para picar en esos momentos. Algunas ideas son: fruta con su cáscara cortada en pedacitos, tomate cortado con queso, pasas de uva. ¡Eso va a mejorar tu alimentación! | DELICIOUS AND HEALTHY FOOD. If you're hungry between meals, you can prepare healthy snacks to munch on during those times. Some ideas include fruit with its skin cut into small pieces, sliced tomato with cheese, or raisins. This will improve your diet. |
| **20** | COMIDA RICA Y SALUDABLE. Si te gusta tomar mate, consulta con tu médico cómo endulzarlo. Podés agregarle edulcorante. | DELICIOUS AND HEALTHY FOOD. If you like to drink mate, consult with your doctor on how to do it. You can add sweetener. |
| **21** | Algunos de los controles necesarios durante el embarazo son: medición de tu peso, altura y presión arterial; escuchar los latidos del bebé; análisis de orina; una prueba para detectar diabetes del embarazo y para enfermedades infecciosas. Anotá todas tus preguntas antes de la consulta para charlarlas con el equipo de salud que te atiende. | Some of the necessary check-ups during pregnancy include: measuring your weight, height, and blood pressure; listening to the baby's heartbeat; performing urine tests; and conducting a test to detect gestational diabetes and infectious diseases. Write down your questions before the consultation to discuss them with your healthcare team. |
| **22** | ¿Sabías que los controles durante tu embarazo son fundamentales para el desarrollo del bebé y detectar y prevenir complicaciones? Un control importante en este trimestre es el análisis para detectar si tenés diabetes del embarazo. ¿Vos ya te lo hiciste? | Did you know that the check-ups during your pregnancy are essential for your baby's development and for detecting and preventing complications? An important check-up during this trimester is the test to detect whether you have gestational diabetes. Have you had it done yet? |
| **23** | Preguntale al equipo de salud que te está atendiendo cuáles son los signos de alarma en el embarazo y cuándo tenés que ir urgentemente al hospital. | Ask your healthcare team what the warning signs during pregnancy are and when you need to go to the hospital urgently. |
| **24** | Moderá el consumo de azúcar y dulces. Evitá las bebidas azucaradas y las gaseosas | Moderate your consumption of sugar and sweets. Avoid sugary drinks and sodas. |
| **25** | Las frutas y las verduras son muy importantes. Tratá de consumirlas todos los días y de diferentes colores, así te aseguras de recibir una mayor variedad de vitaminas y minerales. | Fruits and vegetables are very important. Try to eat fruits every day and of different colors, to ensure you receive a greater variety of vitamins and minerals. |
| **26** | Mediante los controles y análisis, el personal de salud puede detectar algunas enfermedades que pueden afectarlos a vos y a tu bebé, como por ejemplo diabetes del embarazo o hipertensión. Por eso es importante que asistas a todos los turnos. | Through check-ups and tests, healthcare personnel can detect some conditions that might affect you and your baby, such as gestational diabetes or hypertension. That’s why it’s important to attend all your appointments. |
| **27** | ¿Sabías que vos y tu pareja, o la persona que elijas para que te acompañe en el parto, pueden participar de un Curso de Preparación para la Maternidad? ¡Es gratuito en hospitales y CAPS! Averiguá dónde podés hacerlo. | Did you know that you and your partner, or the person you choose to accompany you during childbirth, can participate in a Maternal Preparation Course? It’s free in hospitals and primary health care centers! Find out where you can do it. |
| **28** | La actividad física realizada regularmente puede ayudarte a reducir las molestias del embarazo, como el dolor de espalda, los calambres en las piernas, el estreñimiento, los pies hinchados. | Regular physical activity can help you reduce pregnancy discomforts, such as back pain, leg cramps, constipation, and swollen feet. |
| **29** | La actividad física realizada regularmente durante el embarazo puede ayudarte a controlar los niveles de azúcar en sangre. Preguntale al equipo de salud qué tipo de actividad física podés hacer. | Regular physical activity during pregnancy can help you control your blood sugar levels. Ask your healthcare team what type of physical activity you can do. |
| **30** | Durante el embarazo disfrutá de tomar agua y jugos frutales. Evitá todo tipo de bebidas alcohólicas. | During pregnancy, enjoy drinking water and fruit juices. Avoid all types of alcoholic beverages. |
| **31** | No olvides llevar el Carné Perinatal cuando te internes. Tiene información muy importante y necesaria para atenderte. | Don’t forget to bring your Perinatal Card when you check in. It contains very important and necessary information for your care. |
| **32** | ¿No comés nada y te sentís llena? Es algo normal, ya que tu bebé está más grande y ocupa más lugar! Probá comiendo más veces al día porciones más pequeñas, esto te ayudará a sentirte mejor. | Not eating anything and feeling full? That’s normal, since your baby is bigger and takes up more space! Try eating smaller portions more frequently throughout the day; this will help you feel better. |
| **33** | Es importante comer saludablemente. Controlá el consumo de algunos alimentos, como por ejemplo azúcar, miel, chocolates, caramelos, dulce de leche, helados y gaseosas. Intentá consumirlos solo en ocasiones especiales. | It’s important to eat healthily. Monitor your consumption of certain foods, such as sugar, honey, chocolates, candies, dulce de leche, ice cream, and sodas. Try to consume them only on special occasions. |
| **34** | ¿Te olvidás de tomar agua durante el día? Una buena opción puede ser tener una botella pequeña e ir recargándola a medida que se acabe. | Do you forget to drink water during the day? A good option might be to keep a small water bottle with you and refill it as it runs out. |
| **35** | ¿Sabías que... no es cierto que tomar mucha agua puede provocar que se rompa la bolsa? Tomar mucha agua es muy importante durante el embarazo. | Did you know that it's not true that drinking a lot of water can cause your amniotic sac to rupture? Drinking plenty of water is very important during pregnancy. |
| **36** | Es muy importante para vos y para tu bebé que puedas dormir al menos 8 horas, pero también que busques momentos de tranquilidad durante el día. ¡Tomate pequeños descansos... recostarse o tomar una ducha de agua caliente son buenas opciones! | It is very important for you and your baby to get at least 8 hours of sleep, but also to take moments of calm during the day. Take short breaks—lying down or taking a hot shower are good options. |
| **Final** | Hemos llegado al final de nuestro programa de mensajes. Recordá que siempre contás con el apoyo del equipo de salud de tu centro. | We have reached the end of our message program. Remember that you always have the support of your health center's healthcare team. |
