# Supplementary material for: WhatsApp-Based Intervention for Diabetes Prevention and Care in Argentina: Implementation and Process Evaluation
Source: JMIR Form Res. 2025 Dec 1;9:e81098. doi: 10.2196/81098 (PMC12706442; doi:10.2196/81098)
Supplement: Multimedia Appendix 3 [file formative_v9i1e81098_app3.docx]

**Table S1.** Sociodemographic characteristics associated with intervention acceptance (opt-in).

| **Characteristics** | **OR (CI 95%)** | **p-value ^c^** |
| --- | --- | --- |
| Sex |  |  |
| Female | 1,00 | - |
| Male | 0.84 (0.77–0.93) | <0.0001 |
| Age (per years) | 0.99 (0.98–0.99) | <0.0001 |
| Educational Level^a^ |  |  |
| Primary school | 1,00 | - |
| Secondary school | 1.14 (1.05–1.25) | 0,003 |
| University or higher | 1.58 (1.36–1.84) | <0.0001 |
| Comorbidities^b^ |  |  |
| None | 1,00 | - |
| One condition | 1.22 (1.1–1.35) | <0.0001 |
| Two or more conditions | 1.71 (1.54–1.9) | <0.0001 |

^a^ Includes complete and incomplete levels.

^b^ Includes obesity, hypertension, hypercholesterolemia, and cardiovascular disease.

^c^ p-values < 0.05 indicate statistical significance.
