# Supplementary material for: WhatsApp-Based Intervention for Diabetes Prevention and Care in Argentina: Implementation and Process Evaluation
Source: JMIR Form Res. 2025 Dec 1;9:e81098. doi: 10.2196/81098 (PMC12706442; doi:10.2196/81098)
Supplement: Multimedia Appendix 4 [file formative_v9i1e81098_app4.docx]

**Table S1.** Participant characteristics by opt-in message acceptance and target group.

|  | **Diagnosed diabetes** | | **Risk of diabetes** | | **Pregnant women** | |
| --- | --- | --- | --- | --- | --- | --- |
| **Characteristics, % (n)** | **Opt-in message accepted** | | **Opt-in message accepted** | | **Opt-in message accepted** | |
|  | **Yes** | **No^c^** | **Yes** | **No^c^** | **Yes** | **No^c^** |
| N | 2,169 | 749 | 2,935 | 1,789 | 645 | 251 |
| Female | 68.7% (1,490) | 63.8% (478) | 81.1% (2,380) | 74.2% (1,328) | - | - |
| Male | 31.3% (679) | 36.2% (271) | 18.9% (555) | 25.8% (461) | - | - |
| Age in years, mean (SD) | 50.7 (12.8) | 53.7 (14.5) | 42.8 (13.6) | 46.6 (15.2) | 26.4 (6.0) | 26.4 (5.8) |
| Educational Level^a^ |  |  |  |  |  |  |
| Primary school | 43.1% (934) | 51.9% (389) | 30.4% (893) | 36.5% (653) | 16.9% (109) | 25.1% (63) |
| Secondary school | 46.4% (1,007) | 39.9% (299) | 56.1% (1,647) | 52.1% (932) | 72.7% (469) | 66.9% (168) |
| University or higher | 8.5% (185) | 4.8% (36) | 11.1% (326) | 8.3% (148) | 9.3% (60) | 7.2% (18) |
| Not available | 2.0% (43) | 3.3% (25) | 2.4% (69) | 3.1% (56) | 1.1% (7) | 0.8% (2) |
| Comorbidities^b^ |  |  |  |  |  |  |
| None | 12.4% (270) | 11.9% (89) | 18.0% (529) | 19.3% (346) | 71.2% (459) | 71.7% (180) |
| One condition | 26.0% (565) | 30.2% (226) | 37.8% (1,110) | 38.8% (694) | 24.2% (156) | 23.9% (60) |
| Two or more conditions | 61.5% (1,333) | 57.8% (433) | 44.1% (1,295) | 41.7% (746) | 4.7% (30) | 4.4% (11) |

^a^ Includes complete and incomplete levels.

^b^ Includes obesity, hypertension, hypercholesterolemia, and cardiovascular disease.

^c^ Includes those who declined or did not respond to the opt-in message.
